# Curcumin Administration Improves Force of *mdx* Dystrophic Diaphragm by Acting on Fiber-Type Composition, Myosin Nitrotyrosination and SERCA1 Protein Levels

**DOI:** 10.3390/antiox12061181

**Published:** 2023-05-30

**Authors:** Luisa Gorza, Elena Germinario, Maurizio Vitadello, Irene Guerra, Federica De Majo, Francesca Gasparella, Paolo Caliceti, Libero Vitiello, Daniela Danieli-Betto

**Affiliations:** 1 Department of Biomedical Sciences, University of Padova, 35131 Padova, Italydaniela.danieli@unipd.it (D.D.-B.); 2 Department of Biology, University of Padova, 35131 Padova, Italylibero.vitiello@unipd.it (L.V.); 3 Department of Pharmaceutical Sciences, University of Padova, 35131 Padova, Italy

**Keywords:** curcumin, muscle dystrophy, nNOS, nitrotyrosine, nitrosative stress, oxidative stress, SERCA, AMPK, myotube culture

## Abstract

The vegetal polyphenol curcumin displays beneficial effects against skeletal muscle derangement induced by oxidative stress, disuse or aging. Since oxidative stress and inflammation are involved in the progression of muscle dystrophy, the effects of curcumin administration were investigated in the diaphragm of *mdx* mice injected intraperitoneally or subcutaneously with curcumin for 4–12–24 weeks. Curcumin treatment independently of the way and duration of administration (i) ameliorated myofiber maturation index without affecting myofiber necrosis, inflammation and degree of fibrosis; (ii) counteracted the decrease in type 2X and 2B fiber percentage; (iii) increased about 30% both twitch and tetanic tensions of diaphragm strips; (iv) reduced myosin nitrotyrosination and tropomyosin oxidation; (v) acted on two opposite nNOS regulators by decreasing active AMP-Kinase and increasing SERCA1 protein levels, the latter effect being detectable also in myotube cultures from *mdx* satellite cells. Interestingly, increased contractility, decreased myosin nitrotyrosination and SERCA1 upregulation were also detectable in the *mdx* diaphragm after a 4-week administration of the NOS inhibitor 7-Nitroindazole, and were not improved further by a combined treatment. In conclusion, curcumin has beneficial effects on the dystrophic muscle, mechanistically acting for the containment of a deregulated nNOS activity.

## 1. Introduction

Vegetal polyphenols have long been considered as possible candidates to counteract/delay muscle derangement in Duchenne Muscle Dystrophy (DMD) until efficient and broadly targeted corrective therapies should become available. The rationale to test these compounds depended on their recognized anti-inflammatory and anti-oxidant activities [1] that should antagonize two major pathogenetic players of this genetic disease [2,3].

Although a major feature of vegetal polyphenols is to work as direct Reactive Oxygen Species (ROS) scavengers [4], they can also interfere with various signaling pathways [5]. The non-flavonoid polyphenol from turmeric, curcumin, has been found to interact at a molecular level with several targets [6], among which are inflammatory molecules, cell survival proteins, protein kinases, protein reductases, histone acetyltransferase, histone deacetylase and DNA methyltransferase-1. Binding to the three latter enzymes results in cell-context-specific epigenetic effects, which might explain the curcumin-induced up- or downregulation of a larger variety of proteins, such as transcription factors, enzymes, inflammatory mediators, protein kinases, drug resistance proteins, adhesion molecules, growth factors, receptors, cell cycle regulatory proteins, cell survival proteins, chemokines and chemokine receptors. As a consequence of these indirect and direct interactions, curcumin can prevent dysfunctions of organelles, such as mitochondria [7], and of the autophagic flux [8]. Curcumin supplementation combined with physical activity appears safe, significantly improves the performance of healthy human muscle and reduces exercise-induced oxidative stress [9,10]. Chronic systemic administration of curcumin to laboratory rodents increased lifespan and antagonized loss of muscle mass and force secondary to aging (sarcopenia and presarcopenia) [11,12] or to unloading [13,14]. However, curcumin was not considered among the anti-oxidant approaches that have been tested in the *mdx* mouse model, an animal model for DMD, and been shown to successfully reduce muscle damage and inflammation [3]. In particular, the only two available studies yielded contrasting results. In the first report, a 2-week (wk) diet with 1% curcumin in 3-wk-old *mdx* mice not only failed to switch off the activation of pro-inflammatory transcription in muscles, but also reduced the contractile function of the diaphragm [15]. Conversely, in the second study, positive results were obtained in forelimb and hindlimb muscles by a 10-day systemic administration of curcumin in slightly younger mice (18 days old) [16].

Although the low oral bioavailability of curcumin may explain such a discrepancy and be responsible for the lack of further investigations, the actual efficacy of curcumin against muscle dystrophy progression remains uncertain. The above-mentioned studies investigated an early disease stage, i.e., before the acute bout of cyclic muscle degeneration/regeneration, which begins around 4 wks after birth in the *mdx* mouse model. Furthermore, it has still to be proven whether and how long the reported curcumin beneficial effects on myofiber necrosis, muscle inflammation and force development [16] are detectable in the *mdx* diaphragm, which, at variance with leg muscles, displays a severe dystrophic phenotype that is closer to that found in DMD muscles. The present study was therefore undertaken to evaluate dystrophy progression in the *mdx* diaphragm muscle after systemic curcumin administration that started in 5-wk-old *mdx* mice and lasted for 4, 12 and 24 wks. We addressed the possibility that curcumin would operate through some of the mechanisms that attenuated/antagonized muscle dysfunction secondary to unloading or aging. In those contexts, curcumin reduced oxidative stress levels, maintained the physiological muscle fiber-type composition and/or size, increased protein levels of the Endoplasmic Reticulum (ER) chaperone Grp94 that regulates nNOS subcellular distribution, and enhanced commitment of muscle satellite cells [11,13]. Last but not least, the presence of toxic effects on *mdx* liver secondary to chronic systemic curcumin exposure also has to be assessed.

## 2. Materials and Methods

### 2.1. Mice

Founders from C57BL/10ScSnDmd^mdx^ (*mdx*) and C57BL/10ScSn/OlaHsd (wt) mouse strains were obtained from Charles River Laboratories and Envigo, respectively, and housed and bred in the animal facility of the Vallisneri Biology Building at the University of Padova. Animals had free access to food and water and were kept at a 12 h day–night cycle; cages contained environmental enrichments and accommodated a maximum of 4 adult animals. All experiments were carried out in compliance with national and European bylaws and were approved by the Institutional Ethical Committee of the University of Padova (CEASA).

**Figure 1:**
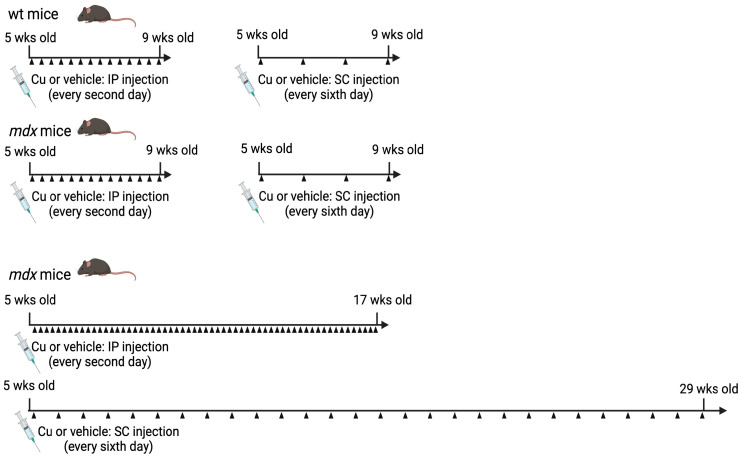
Schematic representation of treatment groups (created with BioRender software (www.biorender.com)).

About 150 five-week-old males were divided into groups to test the effects of systemic curcumin administration, either by intraperitoneal (IP) or subcutaneous (SC) injection, for different treatment times. The IP administration was performed on alternate days and corresponded to the injection of 100 μL of saline, containing 0.1% DMSO and about 40 μg kg^−1^ curcumin, as used for the suspended rat [13]. Curcumin was prepared from a 50 mM stock solution in DMSO and its amount was adjusted every wk accordingly with mean mice body weight, in order to maintain a constant dosage. Control mice were injected with saline added with 0.1% DMSO. The SC administration was performed every sixth day and corresponded to the injection of 100 μL of about 120 μg kg^−1^ of the curcumin formulation described in [11] or an equal volume of vehicle. The experimental groups were: (i) wt mice, which received either IP (*n* = 11) or SC (*n* = 6) curcumin administration for 4 wks, or each one of the two vehicles (*n* = 10 IP + 4 SC); (ii) *mdx* mice, which received either IP (*n* = 17) or SC (*n* = 13) curcumin administration for 4 wks, or each one of the two vehicles (*n* = 14 IP + 12 SC); (iii) *mdx* mice, which received either IP curcumin administration (*n* = 10) for 12 wks, or IP vehicle (*n* = 15); (iv) *mdx* mice, which received SC curcumin administration (*n* = 10) for 24 wks, or SC vehicle (*n* = 16) (Figure 1).

Additional 5-wk-old *mdx* mice were IP injected daily for 4 wks with 50 mg kg^−1^ 7-Nitroindazole (7-NI) in peanut oil, alone (*n* = 9) or in combination with curcumin (*n* = 6), as described in [13].

At the end of each treatment, mice were processed for euthanasia, using isoflurane anesthesia to collect blood by intracardiac puncture, or cervical dislocation for mechanical studies. Seven-month-old wt male mice (*n* = 6) were euthanized without any treatment. Carcasses were weighed, and diaphragm and liver were excised, then either frozen in liquid nitrogen and stored at 80 °C or immediately processed as described below.

### 2.2. Antibodies and Reagents

The following primary antibodies were used: mouse anti-tropomyosin monoclonal antibody (mAb) (clone CH1; 1:200), rabbit anti-nNOS (NOS1) polyclonal antibody (pAb) (1:250), anti-CHOP pAb (1:20) and anti-sarcomeric myosin heavy-chain (MyHC) pAb (1:5000) and goat anti-SERCA1 pAb (1:500) (all from Santa Cruz Biotechnology, Heidelberg, Germany; anti-actin (20–33) rabbit pAb (1:5000) (Sigma-Aldrich-Merck, Milano, Italy); mouse anti-Hsp70 mAb SPA-810 (1:5000, Stressgen); mouse anti-Ki67 mAb (clone MM1, 1:25, Novocastra, Newcastle, UK); rabbit anti-mannose receptor pAb (CD206, 1:1000), anti-dystrophin pAb (1:200) and anti-Grp94 pAb (1:2000) (Abcam, Cambridge, UK); rat anti-F4/80 mAb (clone A3-1, 1:2000, AbDSerotec, BioRad Italia, Segrate, Italy); mouse FITC-conjugated anti-iNOS mAb (1:250, BD Transduction Lab, Firenze, Italy ); goat anti-mouse IGF-I pAb (1:5000) (R&D system, Minneapolis, MN, USA); mouse anti-calsequestrin mAb (clone VIIID12; 1:5000) (Affinity BioReagents, Golden, CO, USA); rabbit anti-Phospho-AMPK mAb (1:1000), rabbit anti-AMPK pAb (1:1500) and anti-cleaved Caspase-3 pAb (1:200) (Cell Signaling, Danvers, MA, USA); rabbit anti-SERCA1 (1:1000) pAb and mouse anti-PMCA mAb (clone 5F10; 1:1000) (Invitrogen-Thermo Fisher Scientific Italy, Segrate, Italy); rabbit anti-sarcolipin pAb (1:500) and anti-nitrotyrosine pAb (1:2000) (Merck-Millipore, Milano, Italy). Mouse anti-Grp94 (clone 3C4; 1:1000) mAb ([17] and Millipore) and mouse anti-embryonic MyHC mAb (clone BF-G6, 1:5000), anti-1MyHC mAb (clone BA-D5; 1:4000), anti-2A MyHC mAb (clone SC-71, 1:1000) and anti-all MyHC types except 2X mAb (clone BF-35 1:1000) were previously described [17,18].

Secondary pAb antibodies conjugated with peroxidase were from Cell Signaling and Dako Cytomation (Glostrup, Denmark). Secondary pAb antibodies conjugated with AlexaFluor-488 or AlexaFluor-568 were from Invitrogen.

### 2.3. Western Blotting

About twenty 10 μm cryosections were collected from the diaphragm muscle in a microtube and homogenized by repeated pipetting in 100 μL of hot Laemmli 2x buffer. After total protein determination [13], equal protein amounts of whole homogenate were separated by polyacrylamide gel electrophoresis (PAGE) in reducing and denaturing 4–13.5% gradient gels and transferred to nitrocellulose in the presence of methanol. Blot strips were incubated, after blocking, with appropriate dilutions of anti-Grp94, anti-Hsp70, anti-CHOP, anti-actin, anti-nNOS, anti-mouse IGF-I, anti-calsequestrin, anti-PMCA, anti-SERCA1, anti-sarcolipin, anti-Phospho-AMPK, anti-AMPK and anti-rabbit, and anti-goat or anti-mouse secondary antibodies conjugated with peroxidase (1:2000). Bound antibodies were revealed using chemiluminescence, as previously described [11], except for the intact actin band, which was revealed using the less sensitive 3,3′,5,5′-Tetramethylbenzidine (TMB, Sigma, Milano, Italy), when investigating its proteolysis. Relative protein levels were calculated by normalization of the densitometric signal to the corresponding amount of loaded serum albumin or actin after staining with Ponceau Red or with TMB in the case of the 14 kDa actin fragment.

For tyrosine nitration analysis, crude myofibrillar extracts were prepared from diaphragm cryostat sections as described in [19]. About 5–10 μg were run on 10–12.5% PAGE gels, transferred onto nitrocellulose and stained with anti-nitrotyrosine pAb or anti-MyHC pAb and anti-rabbit secondary antibodies conjugated with peroxidase (1:2000).

### 2.4. Immunoperoxidase and Routine Histology Stainings

Liver and muscle cryosections (10 μm thick) were collected on gelatin-coated slides and processed for immunoperoxidase with antibodies for cleaved caspase 3, Ki67, CHOP and MyHC isoforms, as previously described [11,17,18], and for hematoxylin–eosin stainings. Azan Mallory and Sirius Red histological stainings were performed to determine the presence of fibrosis. Images were acquired by using an Axioplan optic microscope (Zeiss). Morphometric measurements were made using the ImageJ NIH software version 1.43.

Ki67-positive hepatocyte nuclei were counted on whole cryosections; the percentage of CHOP-positive nuclei was evaluated on more than 600 hepatocytes from three randomly selected fields.

Myofiber regeneration was evaluated by measuring the muscle area occupied by myofibers positive in immunohistochemistry for embryonic MyHC in at least two different cryosections, taken from different sectioning levels, and expressed as percentage of total section area. Centrally nucleated myofibers (i.e., fibers with nuclei distant more than one nuclear diameter from the sarcolemma [20]) were identified with hematoxylin–eosin staining from at least three representative fields and expressed as percentage of the adult myofiber number.

Routine hematoxylin–eosin was used to measure muscle area occupied by mononucleated cell infiltrate from at least two cryosections taken at different sectioning levels and expressed as percentage of total section area. Fibrosis was determined by measuring the blue or red stained area by Azan Mallory or Sirius Red, respectively.

Fiber-type populations were identified by comparing adult myofibers after staining with anti-MyHC antibodies in serial cryosections [18]. At least 400 fibers from two different micrographic fields were considered. Myofiber size was determined by measuring the minimal Feret diameter [11] on at least 60 fibers for each fiber-type population, from two different micrographic fields.

### 2.5. Immunofluorescence

Diaphragm cryosections (10 μm-thick) were collected on gelatin-coated slides and processed for indirect double immunofluorescence with anti-iNOS, anti-CD206, and anti-F4/80 antibodies to identify macrophage subpopulations. Staining was performed according to the manufacturer’s protocol. In brief, cryosections were fixed for 10 min with a drop of 4% paraformaldehyde in phosphate-buffered saline (PBS) at room temperature (RT) and permeabilized and quenched for 1 h at RT in PBS added with 0.1%Tween, 1% bovine serum albumin, 0.3 M glycine and 10% goat serum. The following combinations of antibodies were used: (i) anti-iNOS FITC and anti-CD206, to distinguish M1 from M2 macrophages; (ii) anti-CD206 and anti-F4/80, to evaluate the amount of M2 macrophage population. Serial cryosections were incubated in absence of both or primary antibodies as negative controls. Incubation was performed overnight at 4 C and, after removal of unbound antibodies, cryosections were incubated for 2 h at RT with appropriate dilutions of the secondary antibodies: goat anti-rabbit AlexaFluor-568 (Condition (i)), goat anti-rabbit AlexaFluor-568 and anti-rat AlexaFluor-488 (Condition (ii)). Lipofuscin fluorescence was eliminated by treating sections with Autofluorescence Eliminator Reagent (Chemicon Int. now Merck-Millipore).

Imaging was performed on micrographs collected with the Axioplan microscope equipped with epifluorescence optic (Carl Zeiss, Milano, Italy). For M1 and M2 macrophage counts, about 8 different micrographic fields at high magnification were collected for each diaphragm.

### 2.6. Serum Creatine Kinase (CK) Assay

Blood collected from mice at sacrifice was placed into microtubes and allowed to clot for 30 min at RT. Tubes were then centrifuged for 10 min at 800 g to separate serum. Aliquots of sera were flash frozen in liquid nitrogen and stored at −80 °C. CK assays were performed using Stanbio CK, Liqui-UV (NAC) (BDS International Diagnostic, Germany) and a Perkin-Elmer heated spectrophotometer, following the kit instructions with modifications. In brief, after kit validation by testing human reference sera, kinetics of mouse serum CK were assessed. At variance with the human enzyme, murine CK activity was quickly detectable, even after ten times dilution, and reached saturation after the first minute. Therefore, values reported here derived from the micromolar extinction coefficient of NADP absorptivity at 340 nm measured 15 s and 30 s after addition of serum.

### 2.7. Mechanical Recordings

Force development measurements were performed in vitro in a vertical muscle apparatus (300B, Aurora Scientific Inc., Aurora, ON, Canada) containing a Ringer solution of the following composition: 120 mM NaCl, 4.7 mM KCl, 2.5 mM CaCl_2_, 3.15 mM MgCl_2_, 1.3 mM NaH_2_PO_4_, 25 mM NaHCO_3_, 11 mM glucose, 30 µM d-tubocurarine, pH 7.2–7.4, bubbled with 95% O_2_-5% CO_2_, at 30 °C. Diaphragm strips from central tendon to ribs were dissected, mounted in a vertical bath and stretched to the optimal length; electrical stimulation was supplied by two parallel electrodes, delivering supramaximal pulses (0.5 ms duration) by a Grass S44 electronic stimulator through a stimulus isolation unit (Grass SIU5). Muscle response was recorded through an isometric force transducer (Grass FT03) connected to an AT-MIO 16AD acquisition card (National Instruments). Three strips were dissected for each diaphragm and the mean value was considered for each recorded parameter. At the end of the experiment, strips were weighed, and twitch and tetanic tensions normalized to the wet weight (specific tension, N g^−1^). Time to peak of the twitch (CT) and maximum rate of rise of tetanic tension (Vmax) were also measured. Force–frequency curve was determined by stimulating diaphragm strips at 1, 30, 45, 60, 75, 9, 120, 150 Hz.

### 2.8. Oxyblot and Tropomyosin Oxidation

Protein carbonyl groups were demonstrated using the OxyBlot Protein Oxidation Detection Kit (Chemicon, Merck-Millipore), as previously described [13]. The degree of protein carbonylation was determined after normalization to loaded protein.

Disulfide cross-bridge formation in tropomyosin was investigated by separating SH-blocked crude myofibrillar preparations in both non-reducing and reducing PAGE, followed by immunostaining with the anti-tropomyosin antibody, as previously described [19].

### 2.9. Cell Cultures

C2C12 murine myoblasts (ATCC CRL-1772) and primary satellite cells, isolated from *mdx* hindlimb muscles as described in [11], were used. Briefly, hindlimb muscles were mechanically minced, enzymatically digested to release mononucleated cells and then subjected to cell fractionation via Miltenyi magnetic beads. The myogenic population was then selected by depletion as CD45^−^/CD31^−^/Sca1^−^. Cells were then expanded for 2–3 passages in gelatin-coated plates before being used for subsequent experiments.

For C2C12, about 1.2 × 10^3^ cells per cm^2^ were seeded in 6-well plates and grown in DMEM high-glucose in the presence of 10% fetal bovine serum (FBS). For primary myoblasts, about 0.5 × 10^5^ cells per cm^2^ were seeded on laminin-coated wells in 24-well plates and grown in F12 medium supplemented with 20% fetal bovine serum and 5 ng/mL bFGF. At confluence, myotube formation was induced by switching to the differentiation medium (high-glucose DMEM, 2% horse serum). On day D2, 20 μM cytosine arabinoside (AraC) was added to the differentiation medium to eliminate proliferating cells; the differentiation medium was replaced every 24 h. On day D3, a number of wells were exposed to 1–5 μM curcumin for 3 h as described in [21] and then returned to D medium containing 20 μM AraC. This transient exposure to curcumin was repeated at day D5, and cultures were used at day D6 for biochemical assays.

### 2.10. Statistical Analyses

Data were expressed as mean, median, and 5th–95th percentiles with outliers, when using dot or box-plot representation, and as mean ± SEM, when using histograms or tables. Data were analyzed using the one-way analysis of variance (ANOVA), followed by Newman–Keuls post-hoc test. Unpaired Student’s *t*-test was used when comparing two groups. Values of *p* < 0.05 were considered statistically significant. Analyses were performed using Statistical Package SigmaStat version 2.0 (Systat Software Europe, Frankfurt, Germany).

## 3. Results

### 3.1. Curcumin Treatment of mdx Mice

Effects of curcumin treatment on muscle dystrophy progression were assessed after in vivo administration either by IP or SC injection.

Dosage and frequency of IP curcumin injection or vehicle were validated after 4-wk treatment of 5-wk-old wt mice by monitoring the curcumin-induced increase in Grp94 protein levels [13] in diaphragm muscle homogenates. Curcumin treatment significantly increased Grp94 protein levels (*p* = 0.04), whereas it did not affect Hsp70 protein levels (Figure S1). Dosage and frequency of SC curcumin injection or vehicle in wt mice were previously validated [11].

Groups of 5-wk-old *mdx* male mice were then treated with either curcumin or vehicle by IP injection for 4 and 12 wks or by SC injection for 4 and 24 wks, as indicated in Figure 1. Toxic effects, such as the presence of necrosis, apoptosis, endoplasmic reticulum (ER) stress response and cell proliferation, were investigated in the liver of both wt and *mdx* mice after 4 and 12 wks of IP curcumin treatment and after 24 wks of SC curcumin administration. Signs of necrosis/apoptosis were almost undetectable in both vehicle-treated and curcumin-treated mice (not shown). Hepatocyte proliferation, evaluated by means of Ki67 immunolabeling (Figure S2A), appeared significantly, but transiently, increased in 9-wk-old *mdx* mice compared to wt and older *mdx* mice (ANOVA *p* < 0.001; Figure S2B) and was not affected by curcumin treatment at any experimental point. The ER stress response was investigated by means of the nuclear localization of the transcription factor CHOP (Figure S3A). Although this transcription factor localizes constitutively in the nucleus of a large proportion of hepatocytes, 4-wk curcumin IP injection in wt mice showed a trend to a reduced percentage of CHOP-positive nuclei (Figure S3A), which was consistent with the significant decrease in the total protein amount observed by Western blot analysis (Figure S3B). CHOP nuclear localization in vehicle-treated *mdx* hepatocytes significantly, but transiently, increased in 17-wk-old *mdx* mice compared to 9 and 29-wk-old ones (ANOVA *p* < 0.001). Curcumin did not affect the age-dependent changes in CHOP nuclear localization in the *mdx* liver.

Since muscle necrosis represents a relevant feature of *mdx* mice, a serum creatine kinase (CK) assay was performed on serum samples from different treatment groups (Figure S4A). Serum CK activity of vehicle-treated *mdx* mice appeared very variable but significantly higher than that of wt mice, except for the 29-wk-old *mdx* mice (ANOVA *p* < 0.001). Curcumin treatment did not significantly affect CK activity compared to vehicle-treated *mdx* mice, although *mdx* values after 4-wk-treatment with IP Cu did not differ from wt values. Evaluation of another muscle necrosis index, the 14 kDa actin-cleavage product [22], did not confirm the presence of a curcumin effect on muscle irreversible damage in the 4-week IP and SC treatment groups (Figure S4B).

### 3.2. Effects of Curcumin Treatment on Diaphragm Muscle: Dystrophic Signatures and Fiber-Type Composition

**Figure 2:**
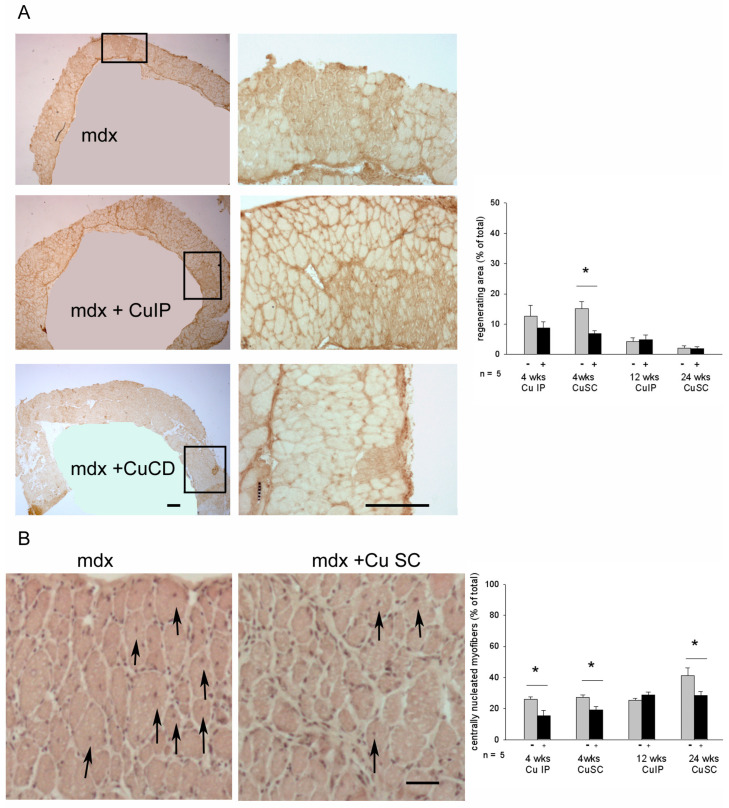
(**A**) Left panels show representative immunostaining with anti-embryonic MyHC antibodies on muscle cryosections from 9-wk-old mdx mice treated for 4 wks with vehicle or with curcumin by means of intraperitoneal (IP) or subcutaneous (SC) administration. Only the diaphragm muscle, which was wrapped onto hindlimb muscles to achieve cryosections for morphological studies, is shown in the low magnification images. The box indicates the field shown at higher magnification. Bars: 200 μm. Right panel shows the histograms of the percentage of diaphragm area occupied by regenerating myofibers in the different treatment groups. Gray and black bars indicate vehicle- and curcumin-treated *mdx* mice, respectively. n: number of mice studied * *p* < 0.05 vs. vehicle-treated mice, Student’s *t*-test. (**B**) Left panels show representative hematoxylin–eosin staining of diaphragm cryosections from 29-wk-old mdx mice treated for 24 wks with vehicle or with SC curcumin. Arrows indicate myofibers displaying central nuclei. Bar: 100 μm. Right panel shows the histograms of the percentage of centrally nucleated myofibers in the different treatment groups. * *p* < 0.05 vs. vehicle-treated mice, Student’s *t*-test.

The regeneration index was evaluated on diaphragm cryosections by measuring the area occupied by small, immunoreactive myofibers for embryonic MyHC [17]; the maturation index was assessed in consecutive serial cryosections by counting centrally nucleated myofibers (Figure 2).

Results for vehicle-treated *mdx* were consistent with literature data [23,24,25], showing a progressive, aging-related decrease in the regeneration index, in the presence of a reduced maturation index. Curcumin significantly decreased the regeneration index only in one instance (4-wk SC treatment), whereas it increased the maturation index by reducing the percentage of centrally nucleated fibers after both IP and SC 4-wk treatments and the SC 24-wk treatment (Figure 2).

**Figure 3:**
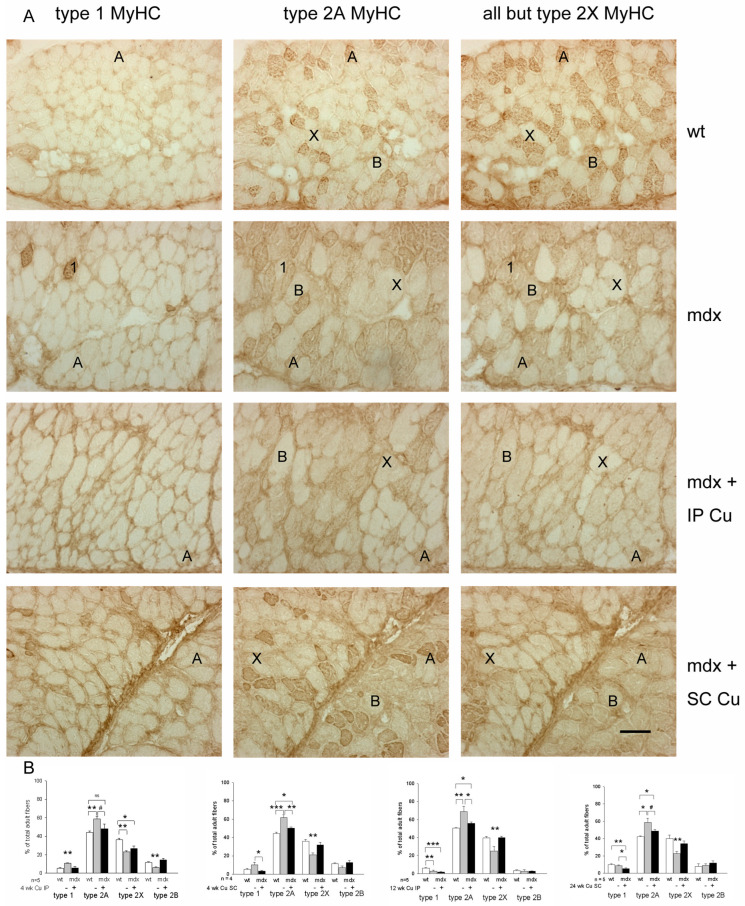
(**A**) Representative immunostaining with isoform-specific anti-MyHC antibodies of diaphragm cryosections from 9-wk-old wt and *mdx* mice, which were treated for 4 wks with vehicle or with curcumin by means of intraperitoneal (IP) or subcutaneous (SC) administration. Type 1 (1) and type 2A (A) fiber types are identified by the presence of dark staining after labeling with the corresponding anti-MyHC antibody. Type 2X (X) fibers are not labeled (light staining) by any of the three antibodies used. Type 2B (B) fibers are not labeled by anti-type 1 and 2A MyHC antibodies, whereas they appear stained by the “all but type 2X” antibody. Bar: 100 μm. (**B**) Histograms of the percentage of each adult fiber type evaluated in the diaphragm of the different treatment groups at the same age. Statistical analyses compared percentages of each fiber-type population among wt (white bar) and vehicle- and Cu-treated *mdx* (gray and black bar, respectively). n: number of mice studied, * *p* < 0.05, ** *p* < 0.01, *** *p* < 0.001, # *p* = 0.08; ns: not significant. ANOVA and post-hoc analyses.

Diaphragm myofiber-type populations were identified via labeling with isoform-specific anti-MyHC antibodies in consecutive cryosections [19], and their relative percentages were calculated without taking into account regenerating myofibers, to allow a better comparison with wt fiber-type composition (Figure 3 and Figure S5).

In the 9-wk-old *mdx* diaphragm, type 1 myofibers appeared increased compared to wt, but their percentage returns were similar to the wt one at older ages (17- and 29-wk-old *mdx* diaphragms), in agreement with [26]. At all the ages studied, the percentage of type 2A myofibers increased significantly in the *mdx* diaphragm compared to wt, whereas type 2X fiber percentage decreased, consistent with the literature [25]. Type 2B fiber percentage appeared unaffected [26], except for the 9-week-old *mdx* diaphragm, where it appeared decreased (Figure 3B).

Curcumin apparently counteracted these changes (Figure 3 and Figure S5). Specifically, the most relevant effect concerned type 2X myofibers, whose percentage was maintained close to the wt value of the corresponding age, after each type of treatment, except for the 4-week IP one. Interestingly, in this same group, treatment was effective in maintaining type 2B fiber percentage at wt level. In addition, curcumin significantly attenuated or displayed a trend to reduce the increase in type 2A fiber percentage, which, however, remained significantly higher compared to wt values. Curcumin treatment also significantly affected type 1 fiber population, which was maintained at wt, or slightly lower ones. In no case did curcumin have an effect on the size of type 2A and 2X myofibers (not shown).

Consistent with the age-related decrease in the regeneration index and literature data [23,25], the inflammation area appeared significantly reduced in the 29-wk-old *mdx* mice compared to the younger mice, whereas fibrosis area significantly increased (Figure S6 and Table 1).

The type of macrophages found in the mdx diaphragm at various ages was also assessed via immunofluorescence (Figure S7 and Table 1). Specifically, M1 macrophages were identified as iNOS^+^ CD206^−^ [27] and M2 macrophages were identified as CD206^+^ F4/80^+^, as described in Methods. Curcumin treatment did not apparently affect the degree of inflammation and the type of macrophage infiltration, nor the deposition of fibrous tissue (Table 1 and Figures S6 and S7).

### 3.3. Effects of Curcumin Treatment on the Contractility of the Dystrophic Diaphragm

**Figure 4:**
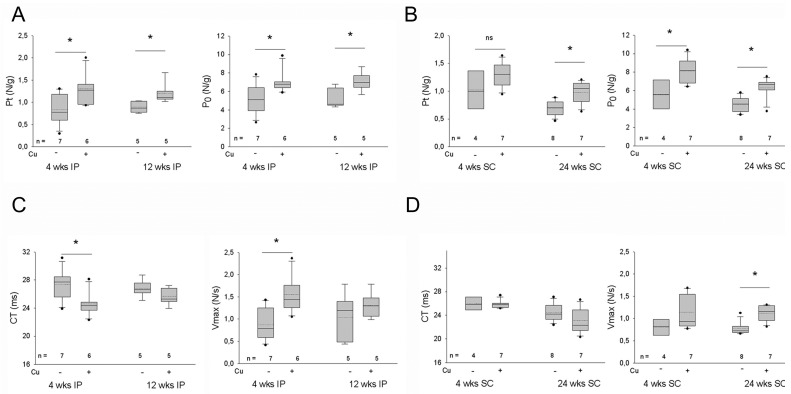
(**A**,**B**) Box plots of twitch (Pt) and tetanic (P_0_) tensions recorded in diaphragm strips of *mdx* mice after intraperitoneal (IP, (**A**) or subcutaneous (SC), (**B**) administration of vehicle or curcumin (Cu) for the indicated weeks (wks). Tension values were normalized to wet weight of diaphragm. Mean and median values are indicated with a dotted and a solid line, respectively. * = *p* < 0.05 (Student’s *t*-test) vs. vehicle values. (**C**,**D**) Box blots of time to peak of the twitch (CT) and maximum rate of rise of tetanic tension (Vmax) in the diaphragm strips from the same mice illustrated in (**A**,**B**), respectively. Mean and median values are indicated with a dotted and a solid line, respectively. * = *p* < 0.05 (Student’s *t*-test) vs. vehicle values.

Mechanical properties of curcumin-treated *mdx* diaphragms were then compared to vehicle-treated *mdx* ones and to diaphragm muscles from wt mice of the same age. Forces developed by *mdx* were significantly lower than those of wt (−55%, not shown). In general, specific twitch and tetanic tensions were significantly responsive to curcumin treatment, since they appeared about 25–30% stronger than those displayed by the vehicle-treated *mdx* diaphragm, although they remained always significantly reduced compared to wt values. The curcumin-induced increase in Pt and Po was evident after each administration route and persisted after long treatment duration when compared with vehicle-treated *mdx* values (Figure 4A,B).

Furthermore, 4-wk IP-treated diaphragm strips showed a transitory reduction of CT and a parallel increase in Vmax (Figure 4C,D). An increased Vmax was also observed after the 24-wk SC treatment.

### 3.4. Curcumin Effects on Myofiber Oxidative and Nitrosative Stress

Since oxidative stress has been hypothesized to affect muscle dystrophy progression [2,3] and curcumin significantly blunted oxidative stress accompanying disuse muscle atrophy [13], we investigated the effect of curcumin administration on myofibrillar protein carbonylation and tropomyosin oxidation in the diaphragm muscle.

Oxyblot assays did not reveal any significant increase in myofibrillar protein carbonylation in 9-wk- and 17-wk-old *mdx* diaphragms compared to wt (Figure S8). Nevertheless, 12 wks of IP curcumin administration significantly reduced carbonylation levels of myofibrillar proteins compared to both wt and vehicle-treated *mdx* diaphragms (ANOVA *p* < 0.02) (Figure S8).

**Figure 5:**
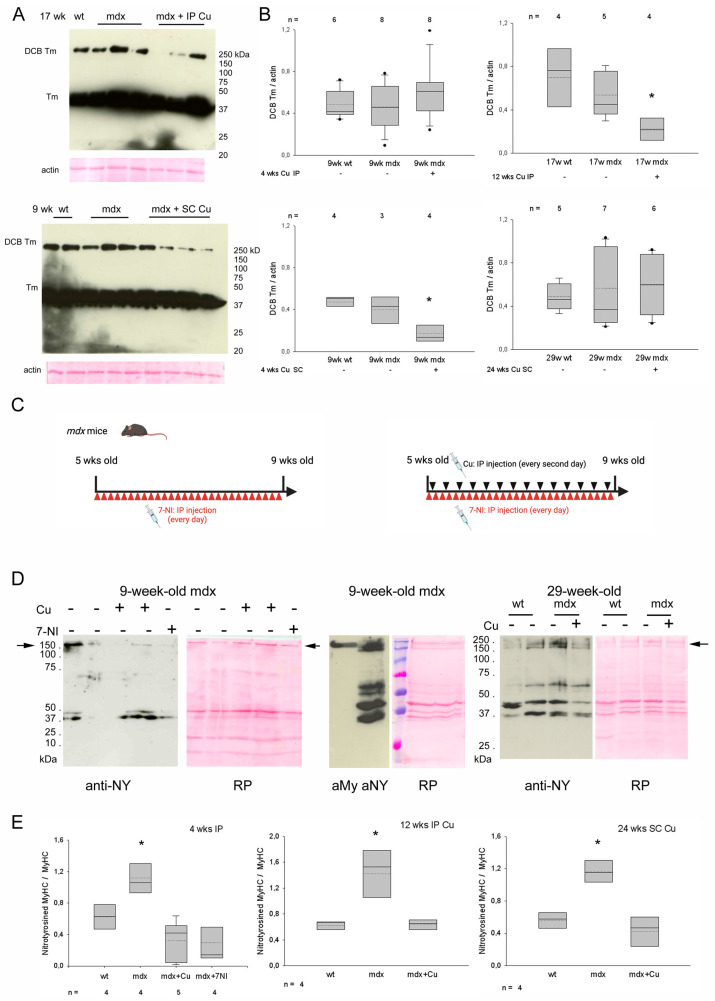
(**A**) Representative anti-Tropomyosin (Tm) Western blot of non-reducing gel electrophoresis of diaphragm homogenates from 17-week-old wild-type (wt) and *mdx* mice, treated for 12 weeks (wks) without or with intraperitoneal (IP) curcumin (Cu) administration (upper panel) and from 9-week-old wt and *mdx* mice, treated for 4 wks with or without subcutaneous (SC) Cu administration (lower panel). DCB Tm indicates polypeptides generated by disulfide cross-bridge formation involving Tm. M_r_ is indicated by molecular weight standards. Loading is indicated by actin staining with Red Ponceau (RP). (**B**) Box plots of DCB Tm levels normalized to actin observed in vehicle- and IP/SC curcumin-treated mice of the indicated age. Mean and median are indicated with a dotted and a solid line, respectively. ANOVA *p* < 0.02. * indicates values significantly different compared to all. n indicates number of analyzed samples in each group. (**C**) Diagram showing the IP treatment protocol of 7-Nitroindazole (7-NI) administration, alone or in combination with Cu (created with BioRender software). (**D**) Left and right panels: representative anti-nitrotyrosine (NY) and RP stainings of Western blots of crude myofibrillar extracts from diaphragms of 9-wk-old mdx mice and 29-wk-old wt and *mdx* mice, treated or not with Cu or 7-NI, as indicated. Middle panel shows the staining with anti-MyHC antibodies (aMy; left) and anti-NY (aNY, right) after cutting in two halves a nitrocellulose blot of a single lane loaded with crude myofibrillar extract from 9-week-old *mdx* diaphragm, as shown by RP staining. M_r_ is indicated by prestained molecular weight standards (from the top: 250, 150, 100, 75, 50, 37, 25 kDa). The arrows indicate MyHC mobility. (**E**) Box plots of NY immunoreactivity detected at MyHC level and normalized to MyHC staining in vehicle- and IP/SC curcumin-treated mice of the indicated age. Mean and median are indicated with a dotted and a solid line, respectively. ANOVA *p* < 0.02. * indicates values significantly different compared to all. n indicates number of analyzed samples in each group.

The apparent lack of increased oxidation levels in the *mdx* diaphragm compared to wt was further investigated by the more sensitive assay of tropomyosin disulfide cross-bridges (DCBs). At variance with what has been reported for leg muscles when comparing mdx to wt mice [28] and independently confirmed by us (not shown), no significant difference occurred in diaphragm tropomyosin oxidation between wt and vehicle-treated *mdx* mice of the same age), consistent with Oxyblot results. Nevertheless, in general, curcumin treatment decreased the tropomyosin oxidation of *mdx* diaphragms (ANOVA *p* < 0.02) (Figure 5A,B).

Nitrosative stress also contributes to loss of *mdx* muscle force [29] and is increased in dystrophic muscles [30]. Thence, we looked for the presence of tyrosine nitration by Western blot analysis of crude myofibrils preparations (Figure 5D,E). Nitrotyrosine immunoreactivity appeared on few polypeptides of about 200, 44 and 37 kDa in diaphragm myofibrillar preparations, corresponding for mobility to MyHC (Figure 5D, middle panel) and presumably to actin and tropomyosin. Interestingly, nitrotyrosine reactivity of MyHC appeared significantly increased in *mdx* compared to wt (Figure 5D), whereas no apparent difference occurred at the level of other polypeptides. The specificity of nitrotyrosine immunoreactivity was validated by the parallel staining of myofibril extracts obtained from *mdx* diaphragms after a 4 wks treatment with the NOS inhibitor 7-NI. In these samples, nitrotyrosine reactivity appeared barely detectable at MyHC level. Curcumin treatment significantly reduced the nitrotyrosine immunoreactivity of MyHC in all the treatment groups (Figure 5D,E).

**Figure 6:**
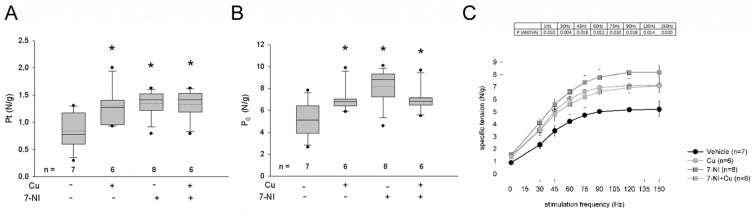
(**A**,**B**) Box plots of twitch (Pt) and tetanic (P_0_) tensions recorded in diaphragm strips of *mdx* mice after 4-wks IP treatment with vehicle or with curcumin (Cu), or/and with 7-Nitroindazole (7-NI). Tension values were normalized to wet weight of diaphragm. Mean and median values are indicated with a dotted and a solid line, respectively. * = *p* < 0.05 (ANOVA and Newman–Keuls post-hoc test) vs. vehicle values. (**C**) Force–frequency relationship measurements in the same samples. Vehicle (black circles), Cu (gray circles), 7-NI alone (dark gray squares) or in combination with Cu (gray squares). Box indicates ANOVA *p* values calculated among points at each stimulation frequency.

To determine whether the decrease in MyHC tyrosine nitration corresponded to an improvement in contractile performance, mechanical investigations were extended to diaphragm strips obtained from mice treated for 4 wks with 7-NI. Results showed an enhanced force development of 7-NI-treated *mdx* mice, consistent with previous findings obtained with the *mdx* EDL muscle [29]. The increase in diaphragm *mdx* force was comparable to that observed after curcumin administration and did not increase further after combined administration (Figure 6).

### 3.5. Curcumin Effects on Protein Levels of Grp94, nNOS and nNOS Regulators

Since we previously identified nNOS as an indirect curcumin target, due to its interaction with the Grp94 chaperone [13], we explored Grp94 and nNOS expression in curcumin-treated *mdx* diaphragms. At variance with what was observed in curcumin-treated wt mice (Figure S1)**,** the 4-wk IP curcumin treatment did not further increase Grp94 protein level in the *mdx* diaphragm; on the other hand, this latter already showed a three-fold increase compared to wt (Figure S9). The possibility that the increase in Grp94 protein level reflected muscle regeneration [18] was supported by the finding of significantly increased levels of IGF-I, a Grp94-client protein, in the vehicle-treated *mdx* diaphragm (Figure S9). Curcumin administration blunted the IGF-I increase, consistent with an enhanced muscle maturation (see Figure 2). Despite the increase in Grp94 protein levels in the *mdx* diaphragm, nNOS protein amount was severely reduced (Figure S9) and localized at sarcolemma only in revertant myofibers (not shown and [31]). Curcumin treatment did not change nNOS subcellular distribution (not shown), nor did it increase its total protein levels (Figure S9). This latter result suggested that curcumin, which was ineffective in improving sarcolemmal nNOS tethering in the *mdx* diaphragm, might nevertheless reduce MyHC tyrosine nitration by inducing sarcoplasmic nNOS inhibition.

**Figure 7:**
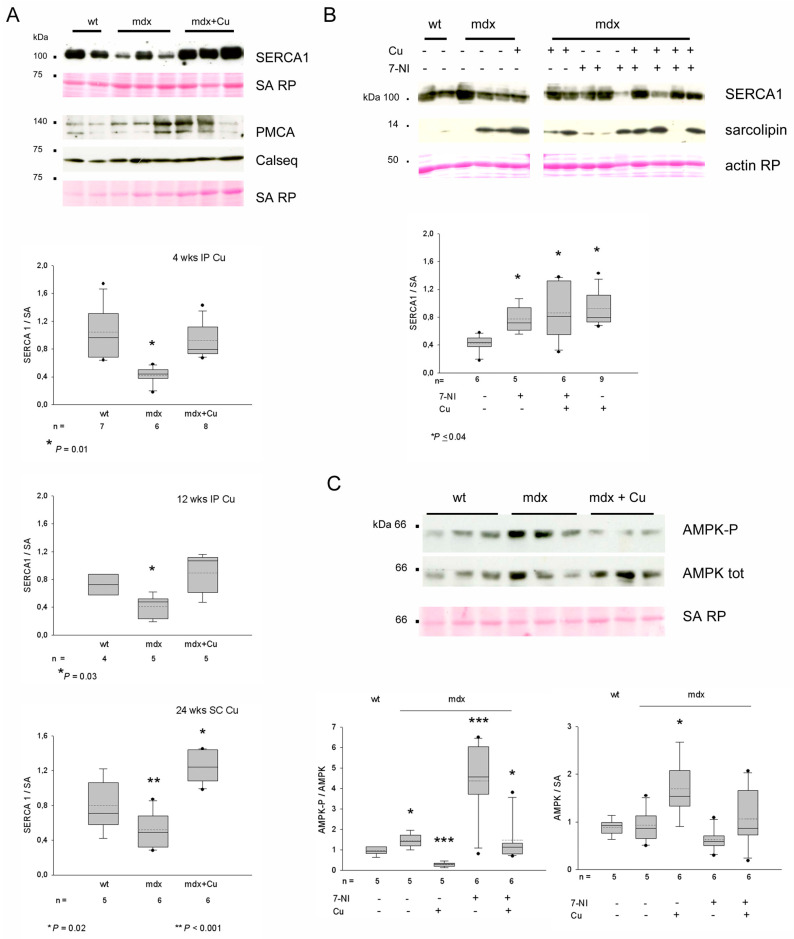
(**A**) Representative Western blots of diaphragm whole homogenates from 17-week (wk)-old wild-type (wt) and *mdx* mice, treated for 12 wks without or with curcumin (Cu) administration and stained with anti-SERCA1, anti-PMCA and anti-calsequestrin antibodies. Loading is indicated by serum albumin (SA) staining with Red Ponceau (RP). Mobility of molecular weight standard is indicated on the left. Box plots show normalized SERCA1 values observed in vehicle- and curcumin-treated mice after different durations and types of treatment. Mean and median are indicated with a dotted and a solid line, respectively. n indicates the number of analyzed samples in each group. * and ** indicate values significantly different compared to all. (**B**) Upper panel: Representative Western blots of diaphragm whole homogenates from 9-wk-old wt and *mdx* mice, treated for 4 wks without or with curcumin (Cu) or/and 7-Nitroindazole (7-NI) and stained with anti-SERCA1 and sarcolipin antibodies. Loading is indicated by actin staining with RP. Mobility of molecular weight standard is indicated on the left. Lower panel: Box plots show normalized SERCA1 values observed in untreated and treated *mdx* mice. Mean and median are indicated with a dotted and a solid line, respectively. n indicates the number of analyzed samples in each group. * indicates values significantly different compared to vehicle-treated mice. (**C**) Representative Western blots of diaphragm whole homogenates from 9-wk-old wt and *mdx* mice, treated for 4 wks without or with curcumin (Cu) and stained with antibodies for phosphorylated and total AMPK. Loading is indicated by SA staining with RP. Mobility of molecular weight standard is indicated on the left. Box plots show values for phosphorylated and total AMPK ratio (left) and normalized total AMPK protein levels (right) observed in wt and untreated and treated *mdx* mice. Mean and median are indicated with a dotted and a solid line, respectively. n indicates the number of analyzed samples in each group. Asterisks (* *p* < 0.03 and *** *p* < 0.001) indicate the presence of significantly different values (ANOVA and post-hoc analyses), as described in the text.

Since calcium represents a major nNOS activator [32], in addition to being a relevant source of damage for dystrophic myofibers [2,33], we investigated whether curcumin treatment affected the expression of proteins involved in calcium storage or cycling (Figure 7).

Figure 7A,B show Western blotting analyses concerning calsequestrin-1, the sarcolemmal- and sarcoplasmic reticulum-calcium pumps PMCA and SERCA1, and the SERCA1-inhibitor sarcolipin [34]. Protein levels of PMCA and calsequestrin did not vary in 9-, 17- and 29-wk-old *mdx* diaphragms compared to wt ones, whereas SERCA1 levels appeared significantly decreased and sarcolipin increased, consistent with previous observations [35,36,37]. Curcumin treatment, independently from the administration route or duration, significantly increased *mdx* diaphragm levels of SERCA1 proteins compared to vehicle-treated *mdx* (ANOVA *p* < 0.03), whereas PMCA and sarcolipin protein levels appeared unaffected. Interestingly, the 4-wk treatment with 7-NI also significantly increased SERCA1 expression in the *mdx* diaphragm (Figure 7B). Curcumin appeared to transiently increase calsequestrin protein levels, since the effect was only seen after the 4-wk IP treatment (ANOVA *p* = 0.05; not shown).

Another pathway involved in the regulation of nNOS activity and a putative target of curcumin is the AMP-Kinase [38,39,40,41,42], whose activation is increased in the *mdx* diaphragm [22]. Our assays confirm the increase in phosphorylated AMPK level in *mdx* compared to wt diaphragms and showed that curcumin significantly reduced the active form while increasing total AMPK protein levels (Figure 7C and Figure S10), independently from treatment duration and way of administration. Conversely, a 4-wk treatment with 7-NI strongly increased AMPK phosphorylation, whereas the combined 7-NI and curcumin treatment maintained it at vehicle-treated *mdx* levels (Figure 7C).

### 3.6. Curcumin Upregulates SERCA1 in Cultured Myotubes

**Figure 8:**
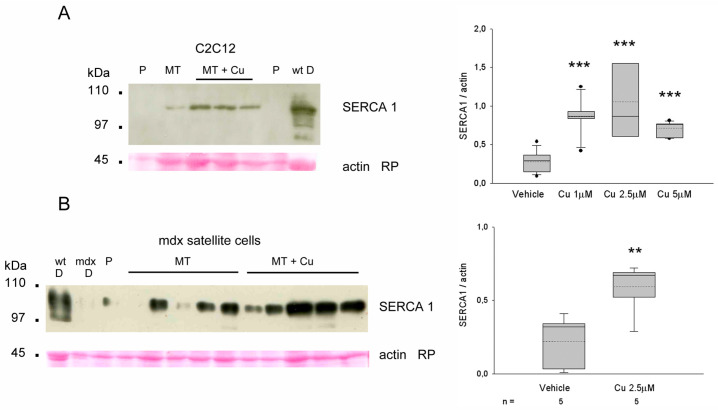
(**A**) Representative Western blot with SERCA 1 antibodies on whole homogenates obtained from cultures of proliferating (P) and differentiated (MT) C2C12 cells, exposed or not to 1 μM curcumin (MT + Cu) as described in Methods, and from wild-type (wt) diaphragm (D) (right panel) Loading is indicated by actin staining with Red Ponceau (RP). Mobility of molecular weight standard is indicated on the left. Box plots show normalized SERCA1 protein levels observed in 6-day differentiating C2C12 MT exposed to vehicle or different Cu dosages (left panel). Mean and median are indicated with a dotted and a solid line, respectively. n indicates the number of analyzed samples in each group. Asterisks (*** *p* < 0.001, ANOVA) indicate the presence of significantly different values compared to vehicle-treated cultures. (**B**) Representative Western blot with SERCA 1 antibodies on whole homogenates obtained from 9-week-old wt and *mdx* diaphragm and from cultures of P and MT *mdx* satellite cells, exposed or not to 2.5 μM Cu (right panel). Loading is indicated by actin RP staining. Mobility of molecular weight standard is indicated on the left. Box plots show normalized SERCA1 protein levels observed in 6-day differentiating MT exposed to vehicle or curcumin dosage (left). Mean and median are indicated with a dotted and a solid line, respectively. n indicates the number of analyzed samples in each group. Asterisks (** *p* = 0.01, Student’s *t*-test) indicate the presence of significantly different values compared to vehicle-treated cultures.

In order to better explore the curcumin effect on SERCA1 protein levels, we investigated myotubes obtained from C2C12 and primary cultures from *mdx* satellite cells, repeatedly and transiently exposed to curcumin, as described in the Material and Methods section (Section 2) and [17] (Figure 8).

C2C12 myotubes showed increased levels of SERCA1 protein compared to proliferating cells, but the exposure to 1–5 μM curcumin upregulated SERCA 1 protein levels about two- to three-fold compared to vehicle-treated myotubes (*p* < 0.001, Student’s *t*-test) (Figure 8A).

Curcumin showed a comparable effect on SERCA1 protein levels when primary myotube cultures obtained by *mdx* satellite cells were exposed to the same treatment protocol (Figure 8B).

## 4. Discussion

This study shows that curcumin treatment significantly attenuated force loss of the *mdx* diaphragm by acting on the fiber-type population and decreasing MyHC tyrosine nitration in the absence of major effects on other dystrophic signatures. In particular, both twitch and tetanic tensions increased by about 30% after curcumin treatment compared to vehicle-treated *mdx* diaphragms. The fiber-type composition of the curcumin-treated diaphragm appeared enriched in the “faster” fiber types 2X and 2B, similar to the wt muscle of the same age. The curcumin-induced decrease in MyHC nitrotyrosination might contribute to the increased force. Our results indicate that such a curcumin effect may be consequent to nNOS inhibition, since the combined treatment with curcumin and the NOS inhibitor 7-NI maintains the positive effect on contractility without showing additional improvements. Apparently, curcumin would negatively affect the activity of the few sarcoplasmic nNOS molecules by acting on both negative and positive nNOS regulators, such as SERCA1 and AMPK-P, respectively. The crucial role played by SERCA1 upregulation in attenuating the loss of force of dystrophic diaphragm has been already highlighted [43,44], and we provide here the first evidence about a chronic, systemic pharmacological treatment that can efficiently increase SERCA1 protein levels in the dystrophic diaphragm.

The systemic administration of curcumin at the dosage used appeared safe, consistent with what was reported after 6-month SC administration in old wt mice [11]. No toxic effect (in terms of apoptosis and ER stress) was observed at any age examined, especially in the liver, which is involved in curcumin catabolism. Such a finding is consistent with the hormetic property of curcumin, which displays beneficial effects at very low dosages, whereas at high dosages, it induces ER stress and apoptosis [6].

As mentioned above, the major and persistent effect of chronic curcumin administration on the *mdx* diaphragm was to reduce by about 30% the loss of force. The increase in tetanic force and the faster contractility are consistent with the changes occurring in fast fiber composition, the lower degree of MyHC tyrosine nitration and lower oxidation of tropomyosin. Moreover, the curcumin-induced increase in SERCA1, by raising the availability of calcium within the sarcoplasmic reticulum, could also contribute to the increase in the force and speed of contraction. In agreement with Petrof et al. [25], the fiber-type composition of 17-wk-old *mdx* diaphragm differs from that of wt mice for the significant increase in type 2A fiber population and the decrease in the type 2X one. Here, we show that comparable changes were observed also for the 29-wk-old *mdx* diaphragm, whereas in the 9-week-old diaphragm, both type 2X and 2B fiber populations appeared reduced. Curcumin treatment counteracted these changes by maintaining the relative percentage of these fiber populations in a proportion comparable to wt values, without affecting the size of fast fibers. Interestingly, we already reported the efficacy of curcumin in maintaining the adult proportion of the type 2X fiber population in the senescent presarcopenic EDL muscle, where the treatment counteracted the reduction in total fiber number that selectively involves this fiber-type population [11]. In fact, we demonstrated that during senescence, curcumin exerts a positive effect on satellite cell commitment [11], a major antagonist of sarcopenia [45] also required for the maintenance of functional neuromuscular junctions [11]. In the *mdx* diaphragm, curcumin treatment did not increase muscle regeneration, but it did reduce the percentage of centrally nucleated myofibers, consistent with the ability to promote muscle maturation [46].

Surprisingly, curcumin did not antagonize inflammation, a major target of vegetal polyphenols [1], but it did maintain its efficacy as an anti-oxidant, as shown here by measuring oxidative changes (protein carbonylation, disulfide bonds) affecting myofibrillar proteins. Strikingly, both approaches did not demonstrate increased myofibrillar oxidation in the *mdx* diaphragm compared to wt at variance with what was described for leg muscles ([28] and our unpublished observations). Nevertheless, the reduction of the oxidative burden operated by curcumin on myofibrillar proteins may lead to beneficial effects and contribute to the increase in contractile performance.

Differently from oxidative stress, nitrosative stress visualized by means of the presence of nitrotyrosine immunoreactivity affected the MyHCs of *mdx* myofibrils more than wt MyHCs and appeared to be significantly reduced by curcumin. Both MyHC and tropomyosin tyrosines have been identified as targets of nitration during aging [47] and in the mechanically ventilated diaphragm [48]. Tyrosine nitration of myosin and other myofibrillar proteins was shown to reduce thin filament velocity and force development in an in vitro motility assay [49]. Here, we show that MyHC tyrosine nitration is counteracted by 7-NI administration, and this effect is consistent with the improvement of the contractile performance of *mdx* muscles after 7NI treatment ([29] and this manuscript). The possibility that 7-NI affected preferentially the activity of the iNOS isoform, which is upregulated in the *mdx* muscle also in the account of inflammation [2,50], has been ruled out by the lack of any improvement in contractile performance observed after iNOS gene ablation [51]. Similarly, the contribution of an inhibitory effect on eNOS seems to be excluded by the evidence that eNOS gene deletion impairs skeletal muscle performance [52]. Interestingly, both 7-NI and curcumin exerted a comparable effect in improving the force of contraction of the *mdx* diaphragm, and the absence of a cumulative effect after the combination of both treatments suggests that they operate through the same pathway. Since both treatments reduced MyHC tyrosine nitration, the possibility exists that curcumin acted as an nNOS inhibitor as well.

Curcumin’s effects on nNOS regulators such as SERCA1 and AMPK-P are consistent with such a hypothesis. In fact, SERCA1 can inhibit nNOS by reducing the cytosolic calcium availability and activity of calmodulin-dependent kinases, whereas AMPK-P would activate nNOS in striated muscles through direct and indirect phosphorylation [38,39,42]. In the *mdx* diaphragm, the reduced levels of SERCA1 protein and the increased activity of AMPK-P might enhance the active state of the nNOS enzyme that accumulates in the sarcoplasm because of the absence of dystrophin. Such a localization might also favor the uncoupling of the nNOS oxidoreductase and foster the generation of nitroperoxides [53]. Curcumin, by significantly increasing SERCA1 protein and decreasing AMPK activation, in spite of a concomitant increase in total AMPK protein, apparently operates in reducing nNOS active state. Interestingly, the direct inhibition of nNOS by 7-NI not only increased further AMPK activation, but also SERCA1 protein levels. Whereas the former effect could be tentatively explained as a feedback response, generated by nNOS inhibition and increased AMP availability secondary to ATP utilization by a less nitrotyrosinated myosin, the latter effect of 7-NI on the increase in SERCA1 protein levels remains obscure.

Conversely, it might be suggested that the upregulation of SERCA1 by curcumin is consequent to the drug-inhibitory effect on the pump [54], which becomes stabilized in the E1 calcium binding form, but unable to bind ATP. It has to be mentioned that such an inhibition was demonstrated in vitro at curcumin concentrations higher than those used here in vivo. Alternatively, SERCA1 upregulation might simply reflect the curcumin-induced preservation of the type 2X myofiber population. Here, we showed that curcumin increased SERCA1 levels in differentiating C2C12 cells and primary *mdx* myotubes, suggesting a direct effect of the drug on this gene expression. Curcumin-induced changes in SERCA1 protein levels were already observed with muscle-specific differences in the senescent hindlimb [11]. At variance with SERCA1, neither 7-NI nor curcumin reduced the levels of the inhibitor sarcolipin, whose upregulation characterizes the dystrophic muscle [36].

## 5. Study Limitations and Conclusions

Our data show that chronic systemic curcumin administration significantly improved the force of the dystrophic diaphragm in the absence of inflammation and/or fibrosis attenuation. Mechanistically, our data point at the inhibition of sarcoplasmic nNOS deregulated activity as the major target of curcumin-induced increase in SERCA1 protein levels and decreased AMPK activation. The control of intracellular calcium handling might also influence fiber-type composition [55,56], and curcumin-induced preservation of type 2X myofiber population might also be a consequence of its improvement, secondary to the increase in SERCA1 protein levels. However, we showed that curcumin effects on type 2X myofiber population in the senescent EDL occurred in the absence of increased SERCA1 levels [11]. Therefore, other targets, possibly leading to the preservation of innervation, have to be considered to explain the positive curcumin effect on this fast fiber-type population.

A major limitation to the translatability of this study is represented by the choice of systemic curcumin administration. In our opinion, it represented the only way to investigate the presence of positive effects using a low curcumin dosage and avoiding interferences from the gastrointestinal tract. Therefore, our results represent a useful starting point to test oral preparations with higher bioavailability, although the protocol of once-a-week subcutaneous administration of the curcumin formulation might be easily applicable in clinical practice after its validation for human use.

Indeed, chronic curcumin treatment also appears to be highly appropriate for the human disease. Fast glycolytic fibers of the human muscle correspond to the rodent type 2X population [57] and are preferentially affected in DMD muscles [58]. A reduced SERCA1 expression also affects the muscle of DMD patients [59], and corticosteroids, which are still currently in use in therapy, are known to decrease SERCA1 transcript levels [60]. Furthermore, recent data indicate that nitrate supplementation, aimed to compensate for the decreased expression of nNOS, showed no apparent beneficial effect in patients and even detrimental ones in myoblast cultures from *mdx* mice and DMD patients [30,61]. Therefore, the use of a compound such as curcumin that improves muscle force by maintaining type 2X myofiber population, increasing SERCA1 levels and inhibiting nNOS in the dystrophic muscle would be very helpful for DMD patients until gene-correcting therapies become available.

## Figures and Tables

**Table 1:** Effects of curcumin treatment on inflammatory signatures in *mdx* diaphragm.

Age + Treatment (Mice Number)	Type of Treatment	Inflammation (Area, %)	M1 Macrophages (iNOS^+^ CD206^−^, %)	M2 Macrophages (CD206^+^ F4/80^+^, %)	Fibrosis (Area, %)
5 + 4 wks (*n* = 8)	IP vehicle	6.19 ± 1.17	58.50 ± 0.64	51.33 ± 3.34	28.19 ± 3.54
5 + 4 wks (*n* = 6)	IP Cu	7.55 ± 1.67	53.50 ± 0.28	40.00 ± 5.45	30.02 ± 3.72
5 + 12 wks (*n* = 5)	IP vehicle	10.45 ± 1.53	27.50 ± 6.00	50.20 ± 4.49	41.80 ± 7.12
5 + 12 wks (*n* = 9)	IP Cu	9.35 ± 0.80	31.25 ± 5.93	51.16 ± 2.77	40.69 ± 3.67
5 + 24 wks (*n* = 4)	SC vehicle	2.37 ± 0.50	35.87 ± 2.47	42.52 ± 7.94	43.47 ± 1.57
5 + 24 wks (*n* = 6)	SC Cu	1.97 ± 0.30	33.28 ± 3.45	30.00 ± 3.10	46.85 ± 1.74

Percentage of M1 macrophages was calculated on the sum of iNOS^+^ and CD206^+^ cells; percentage of M2 macrophages was calculated on the number of F4/80^+^ cells. Comparisons were performed between vehicle and Cu-treated *mdx* mice of the same age (Student’s *t*-test).

## Data Availability

Data is contained within the present article and supplementary material.

## Supplementary Materials

The following supporting information can be downloaded at: https://www.mdpi.com/article/10.3390/antiox12061181/s1, Figure S1: Curcumin effects on Grp94 and Hsp70 expression in wt diaphragm; Figure S2: Curcumin effects on hepatocyte proliferation; Figure S3: Curcumin effects on hepatocyte CHOP expression; Figure S4: Curcumin effects on necrosis biomarkers in wt and *mdx* mice; Figure S5: Effect of curcumin on adult fiber-type composition of the *mdx* diaphragm; Figure S6: Effect of curcumin on inflammation and fibrosis of the *mdx* diaphragm; Figure S7: Effect of curcumin on macrophage populations in the *mdx* diaphragm; Figure S8: Curcumin effects on diaphragm protein carbonylation; Figure S9: Curcumin effects on diaphragm expression of Grp94-interacting proteins; Figure S10: Curcumin effects on diaphragm AMPK-P and total AMPK protein levels.
