# Supplementary material for: Curcumin Administration Improves Force of mdx Dystrophic Diaphragm by Acting on Fiber-Type Composition, Myosin Nitrotyrosination and SERCA1 Protein Levels
Source: Antioxidants (Basel). 2023 May 30;12(6):1181. doi: 10.3390/antiox12061181 (PMC10295800; doi:10.3390/antiox12061181)
Supplement: Supplementary file 1 [file antioxidants-12-01181-s001.zip › antioxidants-2371807-supplementary.pdf]

Supplemental Files

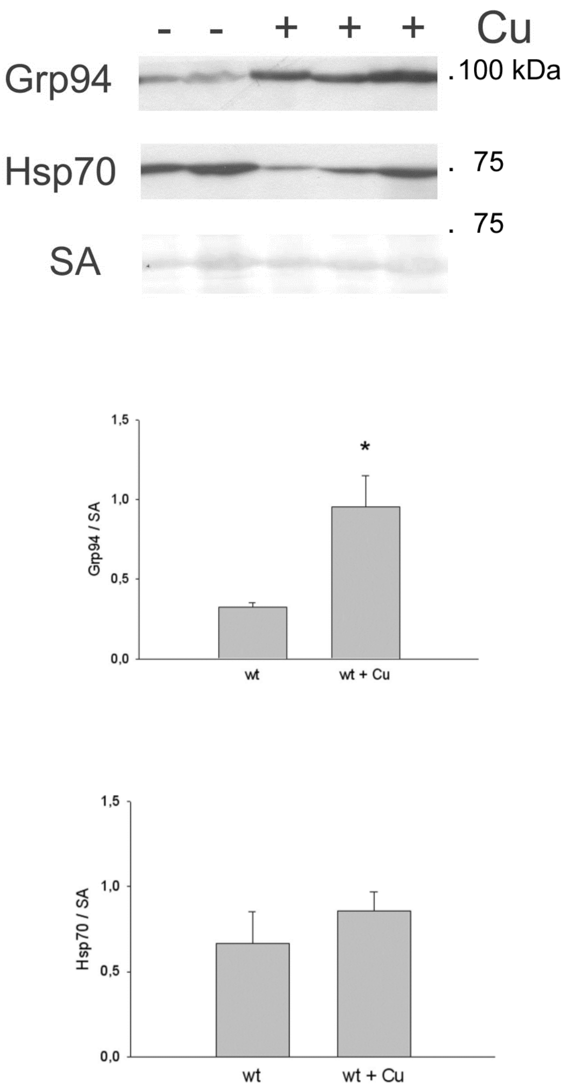

Figure S1: Curcumin effects on Grp94 and Hsp70 expression in wt diaphragm.

Upper panels: Representative Western blot of diaphragm homogenates from 9-week old wild type (wt) mice treated for 4 weeks with (+) or without (-) intraperitoneal curcumin (Cu) administration, stained with anti-Grp94 and –Hsp70 antibodies. Red ponceau staining of serum albumin (SA) is shown as loading reference. Molecular weight standards are shown on the right.

Middle and lower panels: Histograms showing mean and SE values. Number of mice studied for each group: 6. * P=0.04, Student’s t-test

A

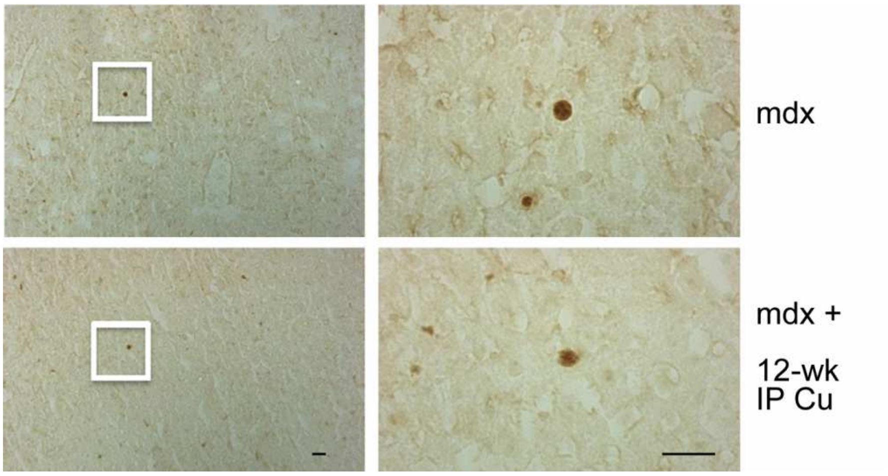

B

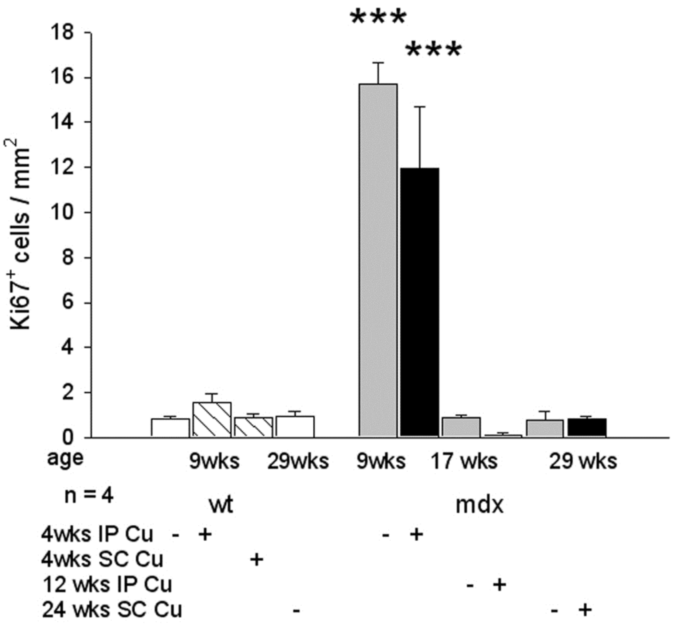

Figure S2: Curcumin effects on hepatocyte proliferation

A: Representative immunostaining of proliferating hepatocytes from vehicle- and curcumin-treated *mdx* mice for the indicated period using anti-Ki67 antibodies. Right panels show a higher magnification of the square areas in left panels. Bars: 100 µm.

B: Histograms showing mean and SE values of $Ki67^+$ hepatocytes, expressed per squared mm. White bars: vehicle-treated wt mice; striped white bars: curcumin-treated wt mice; grey bars: vehicle-treated mdx mice; black-bars curcumin treated mdx mice. n: number of mice analyzed for each grup. ANOVA $P<0.0001$; post-hoc $P<0.001$ vs all, except between vehicle and Cu treated 9wk old mdx samples

A

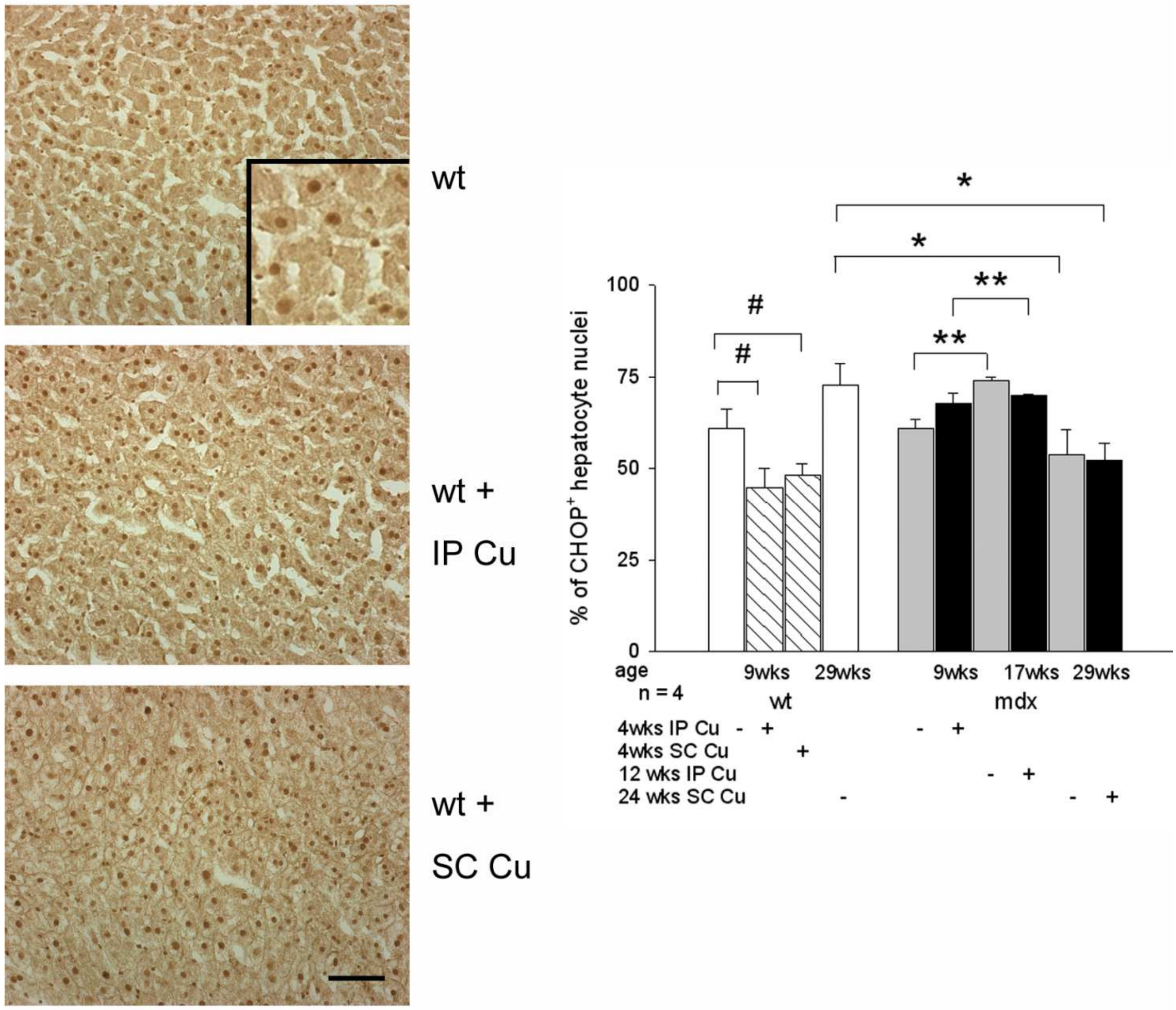
wt
wt +
IP Cu
wt +
SC Cu
% of CHOP+ hepatocyte nuclei
100
75
50
25
0
#
#
*
*
**
**
age
n = 4
9wks
29wks
wt
9wks
17wks
29wks
mdx
4wks IP Cu
4wks SC Cu
12 wks IP Cu
24 wks SC Cu

B

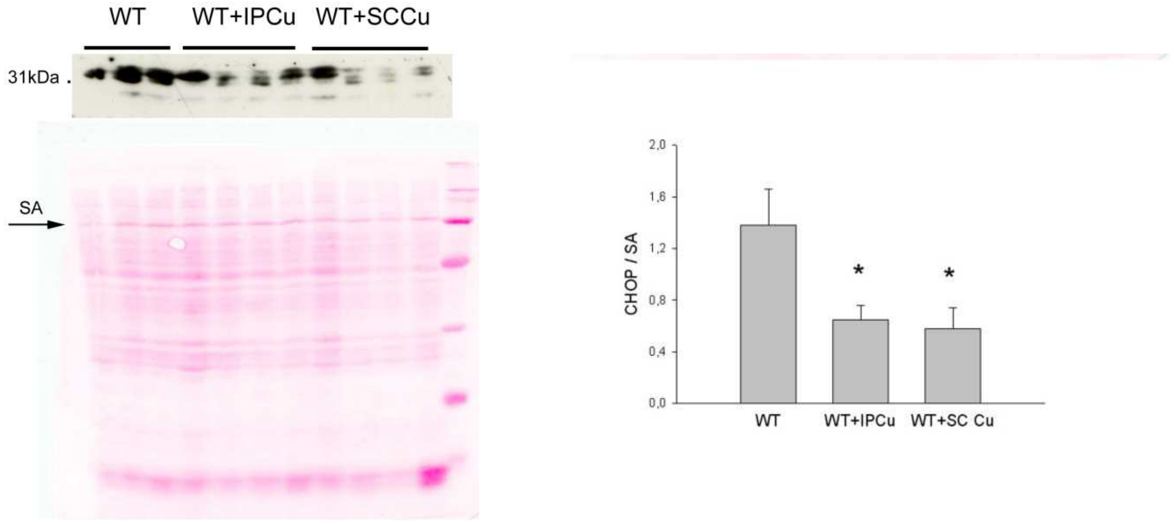
WT
WT+IPCu
WT+SCCu
31kDa
SA
CHOP / SA
2,0
1,6
1,2
0,8
0,4
0,0
*
*
WT
WT+IPCu
WT+SC Cu

Figure S3: Curcumin effects on hepatocyte CHOP expression.

A: Left panels: representative immunostaining with anti-CHOP antibodies on hepatocytes from 9-wk-old *wt* mice after a 4 wk treatment with vehicle (wt) or intraperitoneal (IP) or subcutaneous (SC) injection of curcumin (Cu). Inset shows an higher magnification of the same field. Bar: 100 μm. Right panel: : Histograms showing mean and SE values of CHOP+ hepatocyte nuclei expressed as percentage of total hepatocyte nuclei. White bars: vehicle treated wt mice; striped white bars: curcumin treated wt mice; grey bars: vehicle treated mdx mice; black bars curcumin treated mdx mice. n: number of mice studied for each group. ANOVA $P<0.0001$; post-hoc $^{**}P\leq 0.01$; * $P\leq 0.05$; # P 0.06 between the indicated histograms.

B: Left panels: Representative Western blot of liver homogenates from 9-week old wild type (WT) mice treated for 4 weeks either with vehicle, or intraperitoneal (IP), or subcutaneous (SC) Cu administration, stained with anti-CHOP antibodies and Red Ponceau staining of the same gel with indication of serum albumin (SA) migration as loading reference. Right panel: Histograms showing mean and SE of CHOP densitometric values after normalization with SA. *ANOVA P=0.03

A

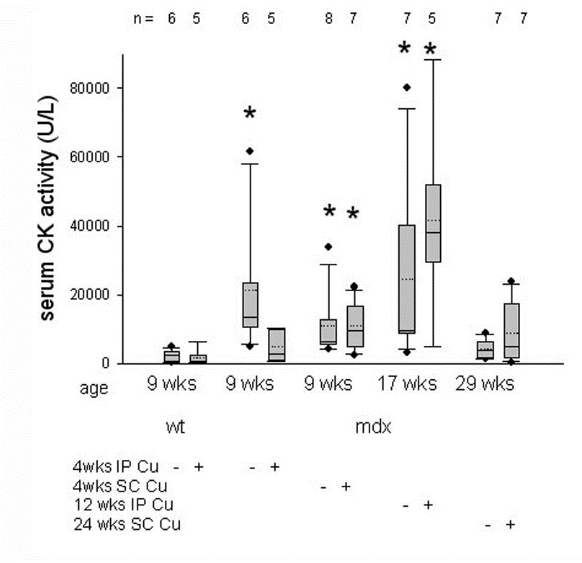

B

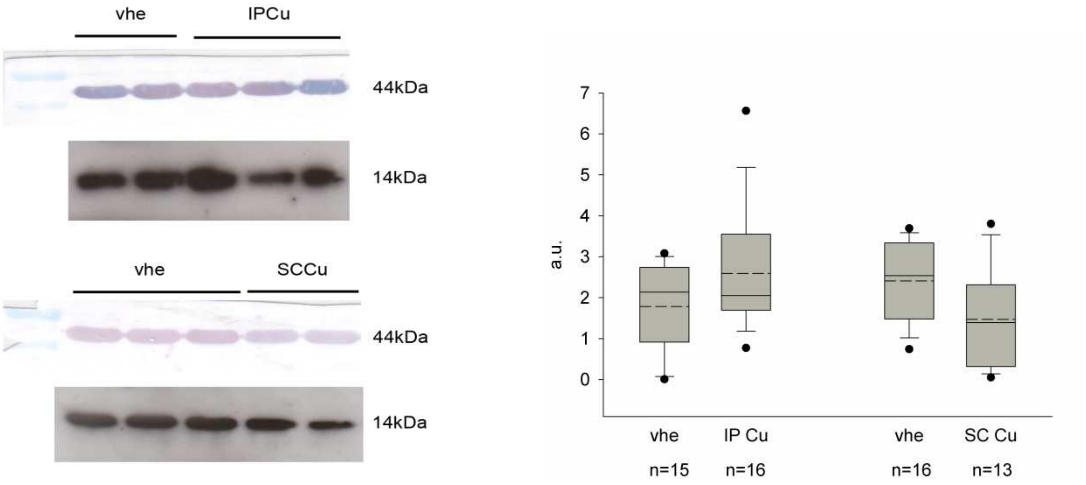

Figure S4: Curcumin effects on necrosis biomarkers in wt and *mdx* mice

A) Box plots of serum CK values for wt and mdx mice of the indicated age after intraperitoneal (IP) or subcutaneous (SC) administration of vehicle or curcumin (Cu) for the indicated weeks (wks). Mean and median are indicated with a dotted and a solid line, respectively. ANOVA $P<0.001$. * indicates values significantly different compared to wt ones. n indicates the number of samples in each group.

B) Left panels: representative Western blots of diaphragm homogenates from 9wk-old mdx mice after 4-wks vehicle-(vhe) or Cu treatment by means of IP or SC administration stained with anti-actin antibody and developed with TMB (44kDa protein) or ECL (14kDa fragment). Right panels: Box plots of actin 14kDa/44kDa ratio Mean and median are indicated with a dotted and a solid line, respectively. n indicates number of analyzed samples in each group.

A

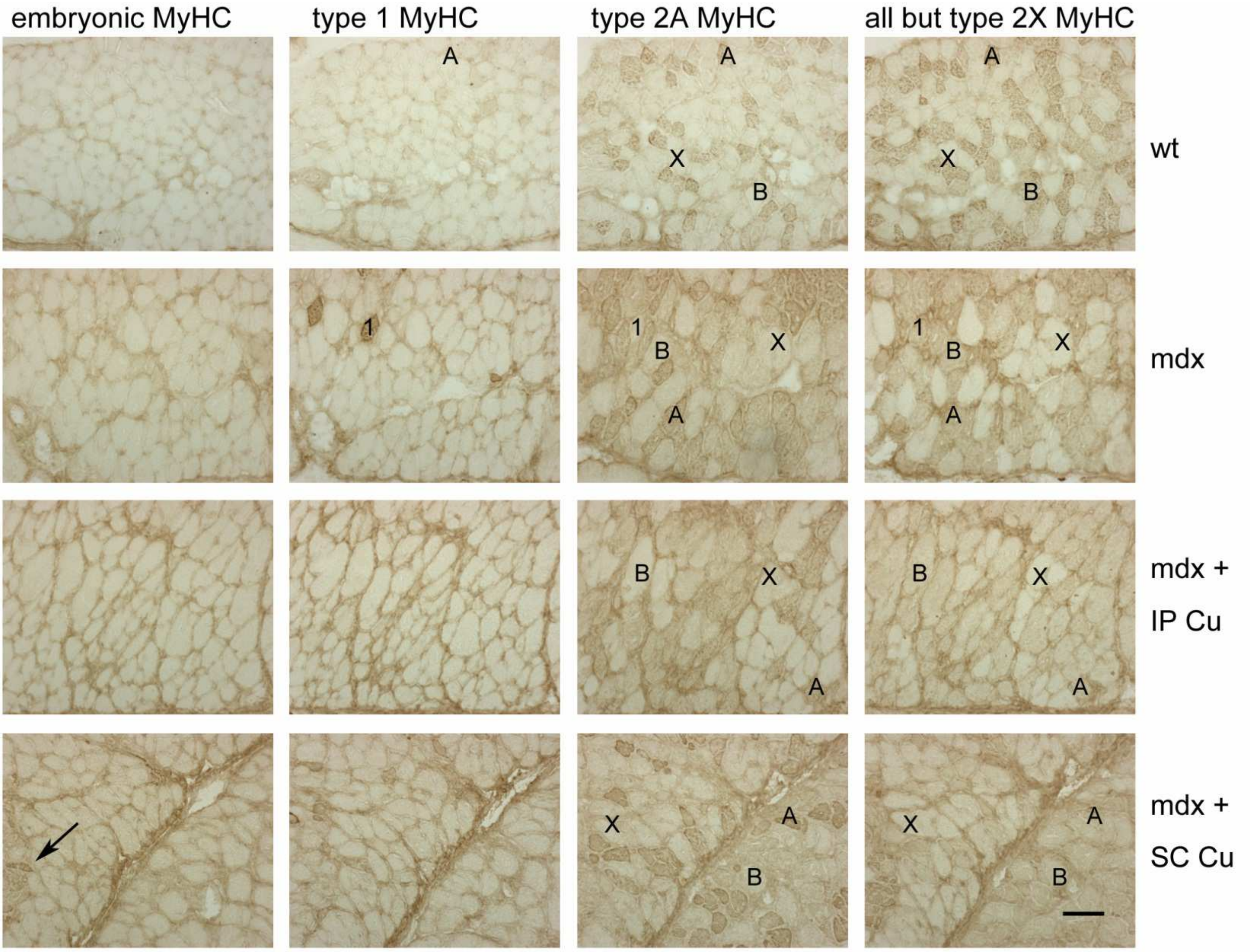

Figure S5A: Effect of curcumin of adult fiber type composition of the mdx diaphragm

Panels show the reactivity for embryonic MyHC in serial fields to those stained for with isoform-specific anti-MyHC antibodies and illustrated in Figure 3. Diaphragm cryosections were from 9-wk-old wt and mdx mice, treated for 4 wks with vehicle or with curcumin (Cu) by means of intraperitoneal (IP) or subcutaneous (SC) administration. Arrow indicates few regenerating myofibers. Bar= 100 μm.

B

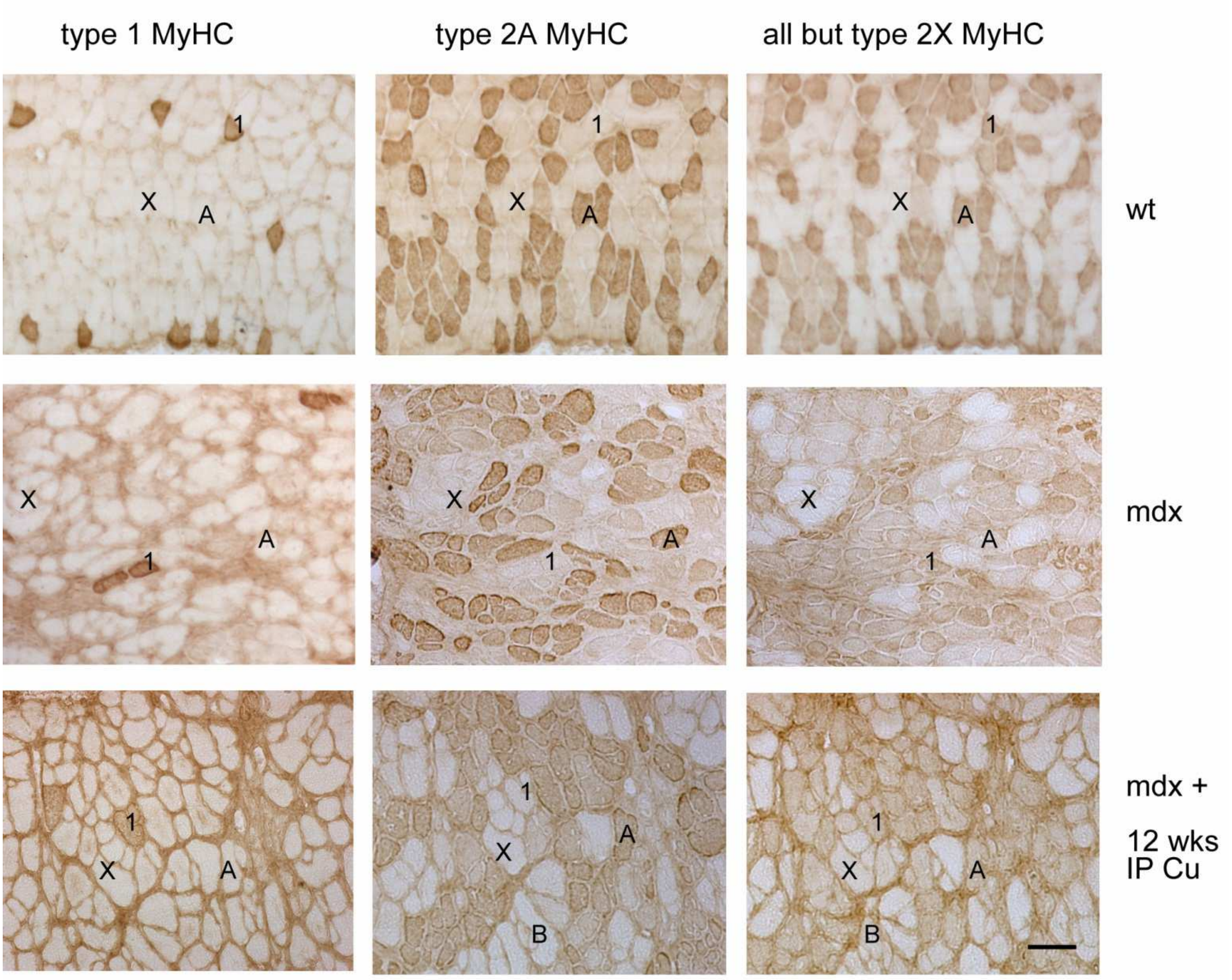

Figure S5B Effect of curcumin of adult fiber type composition of the mdx diaphragm

Representative immunostaining with isoform-specific anti-MyHC antibodies of diaphragm cryosections from 17-wk-old wt and mdx mice, treated for 12 wks with vehicle or with IPCu. Type 1 (1) and type 2A (A) fiber types are identified by the presence of dark staining after labeling with the corresponding anti-MyHC antibody. Type 2X (X) fibers are not labeled by any of the three antibodies used and remain of a lighter color. Type 2B (B) fibers are not labeled by anti-type 1 and 2A MyHC antibodies, but they are stained by the “all but type 2X” antibody. Bar= 100 μm.

C

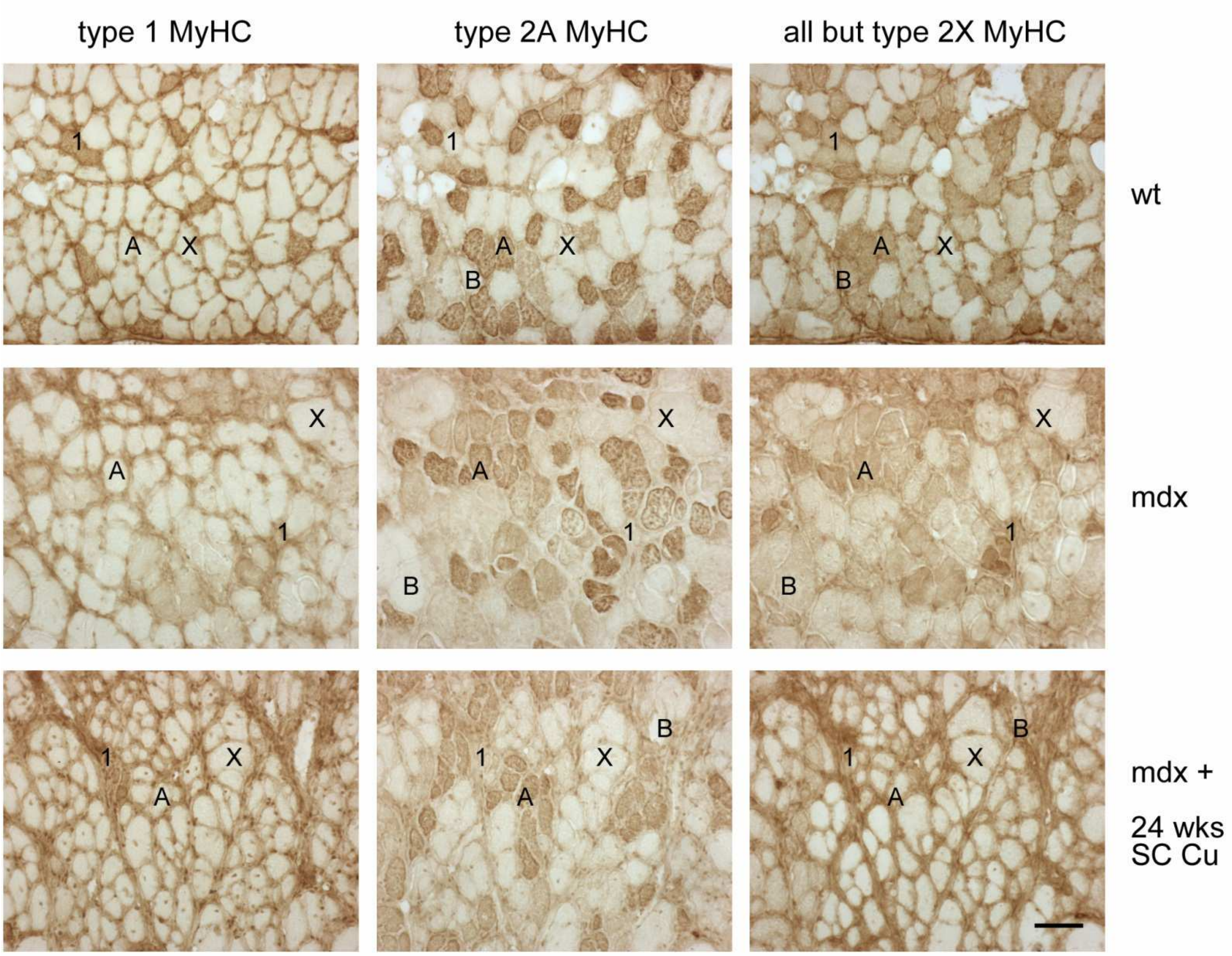

Figure S5C: Effect of curcumin of adult fiber type composition of the mdx diaphragm Representative immunostaining with isoform-specific anti-MyHC antibodies of diaphragm cryosections from 29-wk-old wt and mdx mice, treated for 24 wks with vehicle or SC Cu. Type 1 (1) and type 2A (A) fiber types are identified by the presence of dark staining after labeling with the corresponding anti-MyHC antibody. Type 2X (X) fibers are not labeled by any of the three antibodies used and remain of a lighter color. Type 2B (B) fibers are not labeled by anti-type 1 and 2A MyHC antibodies, but they are stained by the "all but type 2X" antibody. Bar= 100 μm.

A

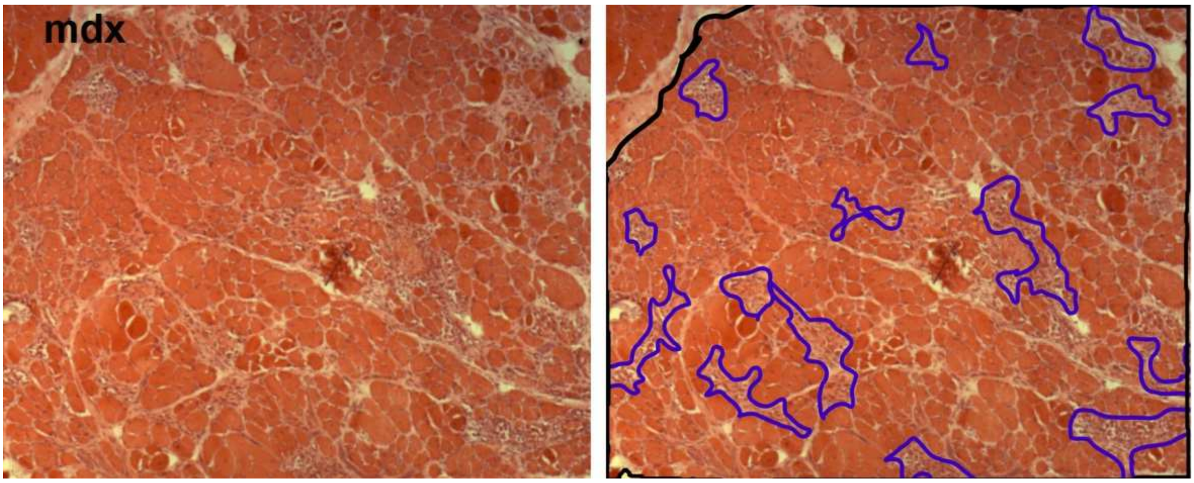

B

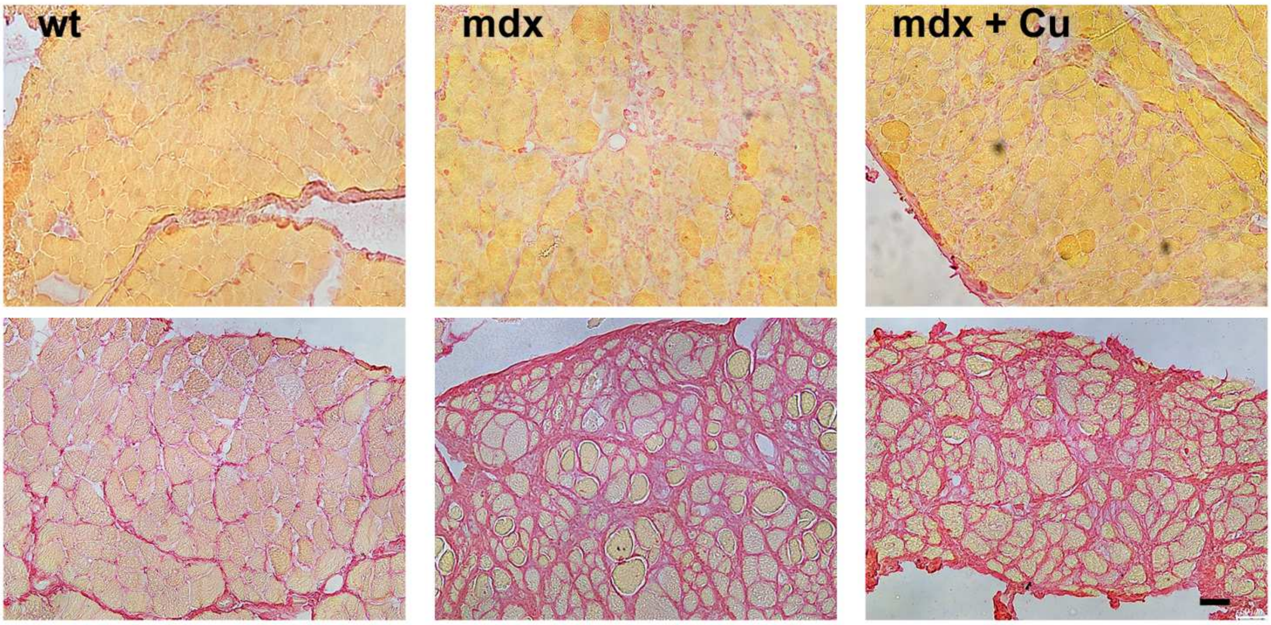

Figure S6: Effect of curcumin on inflammation and fibrosis of the mdx diaphragm.

A) Left panel: representative haematoxylin and eosin staining of a 17-wk-old mdx diaphragm cryosection . The right panel shows the same field where blue lines circumscribe regions containing inflammatory infiltrate and the black line delimitates the useful field boundary.

B) Sirius Red staining of diaphragm cryosections from 9-week (upper row) and 29-week old (lower row) animals. Connective tissue and fibrotic areas are stained in red. Bar: 50μm.

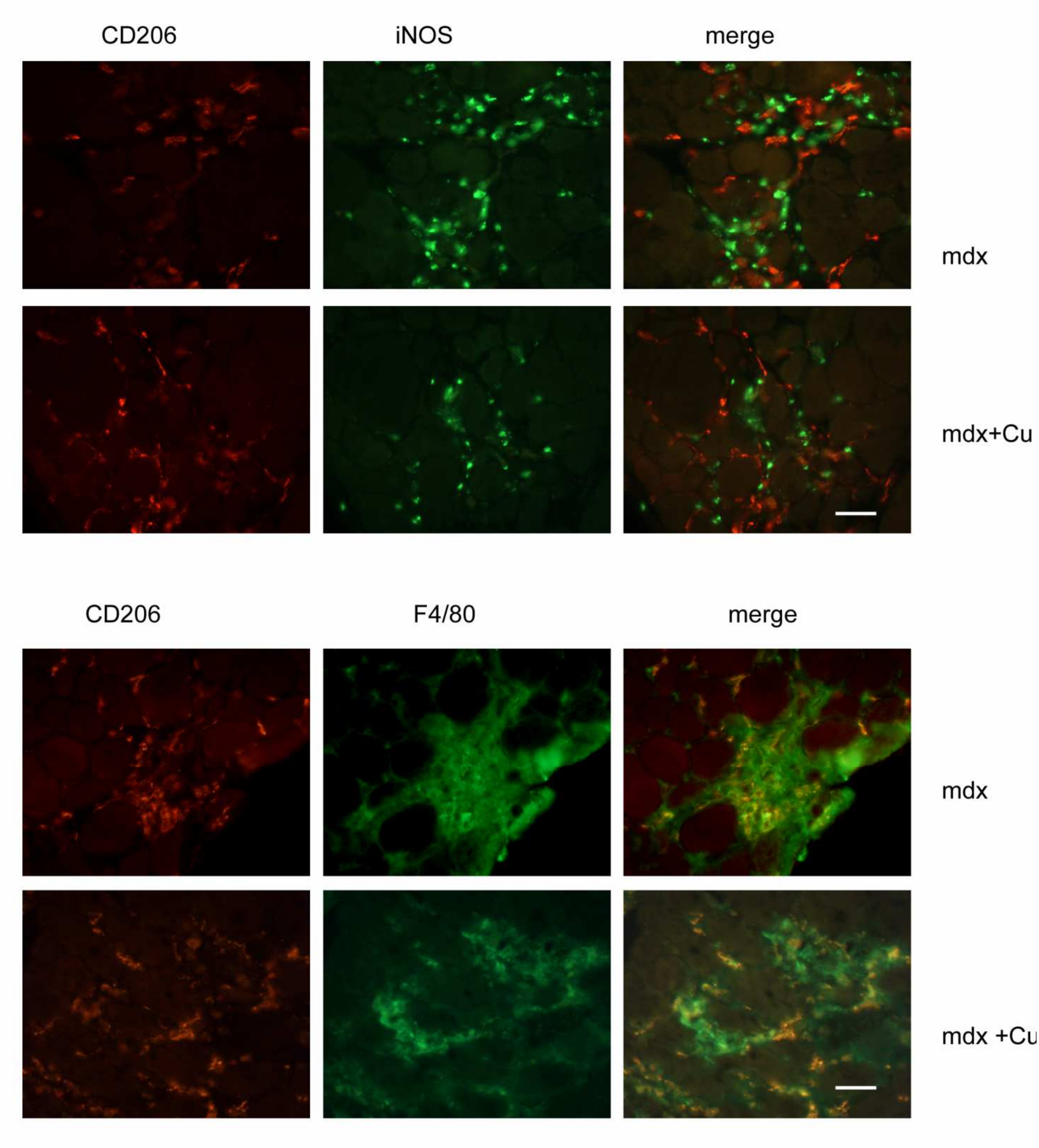

Figure S7: Effect of curcumin on macrophage populations in the mdx diaphragm. Representative double immunofluorescence staining with anti-CD206 antibodies (red fluorescence) combined with anti-iNOS (upper panel) or anti-F4/80 (lower panels, green fluorescence) of inflammatory foci in mdx diaphragm. Merge of both signals (right column) does not show labeling overlap when using CD206 antibodies in combination with iNOS labeling, whereas only a proportion of F4/80 positive macrophages displays also reactivity for the CD206 marker (orange fluorescence). Cryosections were from 29-wk-old wt and mdx mice, treated for 24 wks with vehicle or subcutaneous injection of curcumin (Cu). Bars: 50 µm.

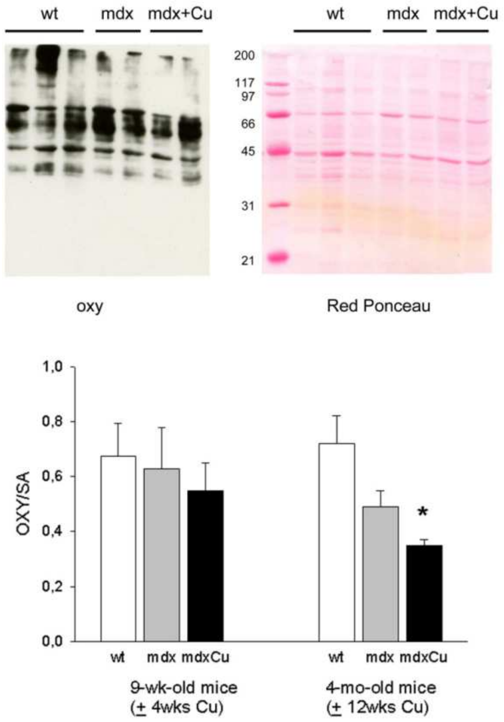

Figure S8: Curcumin effects on diaphragm protein carbonylation

Upper panels: Representative Oxyblot and Red Ponceau stainings of diaphragm homogenates from 19-week old wild type (wt) and mdx mice treated for 12 weeks with vehicle or curcumin (+Cu).

Lower panels: Histograms representing densitometric values of Oxyblot lanes after normalization with serum albumin (SA) staining with Red Ponceau values, evaluated after 4 and 12 weeks of intraperitoneal (IP) treatment with vehicle or curcumin (+Cu) and compared to same age wt mice. *ANOVA P<0.02 vs all

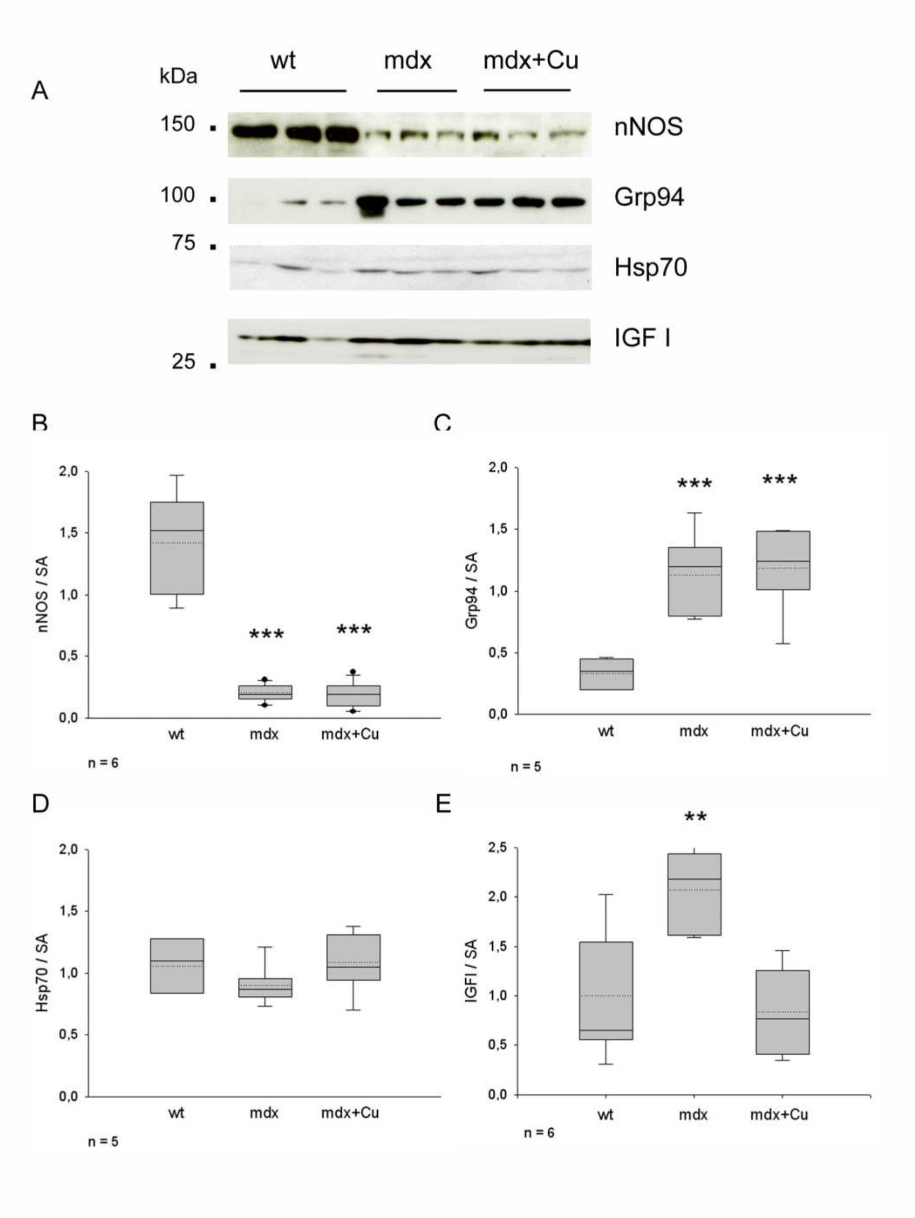

Figure S9 : Curcumin effects on diaphragm expression of Grp94-interacting proteins

A) Representative Western blots of diaphragm homogenates from 9-week old wild type (wt) and mdx mice IP treated for 4 weeks with vehicle or curcumin (+Cu) and stained with antibodies specific for nNOS, Grp94, Hsp70 and IGF-I proteins. Molecular weight standards are indicated on the left.

B– E) show box plots of densitometric values after normalization with serum albumin (SA) stained with Red Ponceau. Mean and median are indicated with a dotted and a solid line, respectively. *** ANOVA *P*<0.001.; ** ANOVA *P*<0.01. n indicates the number of analyzed samples in each group.

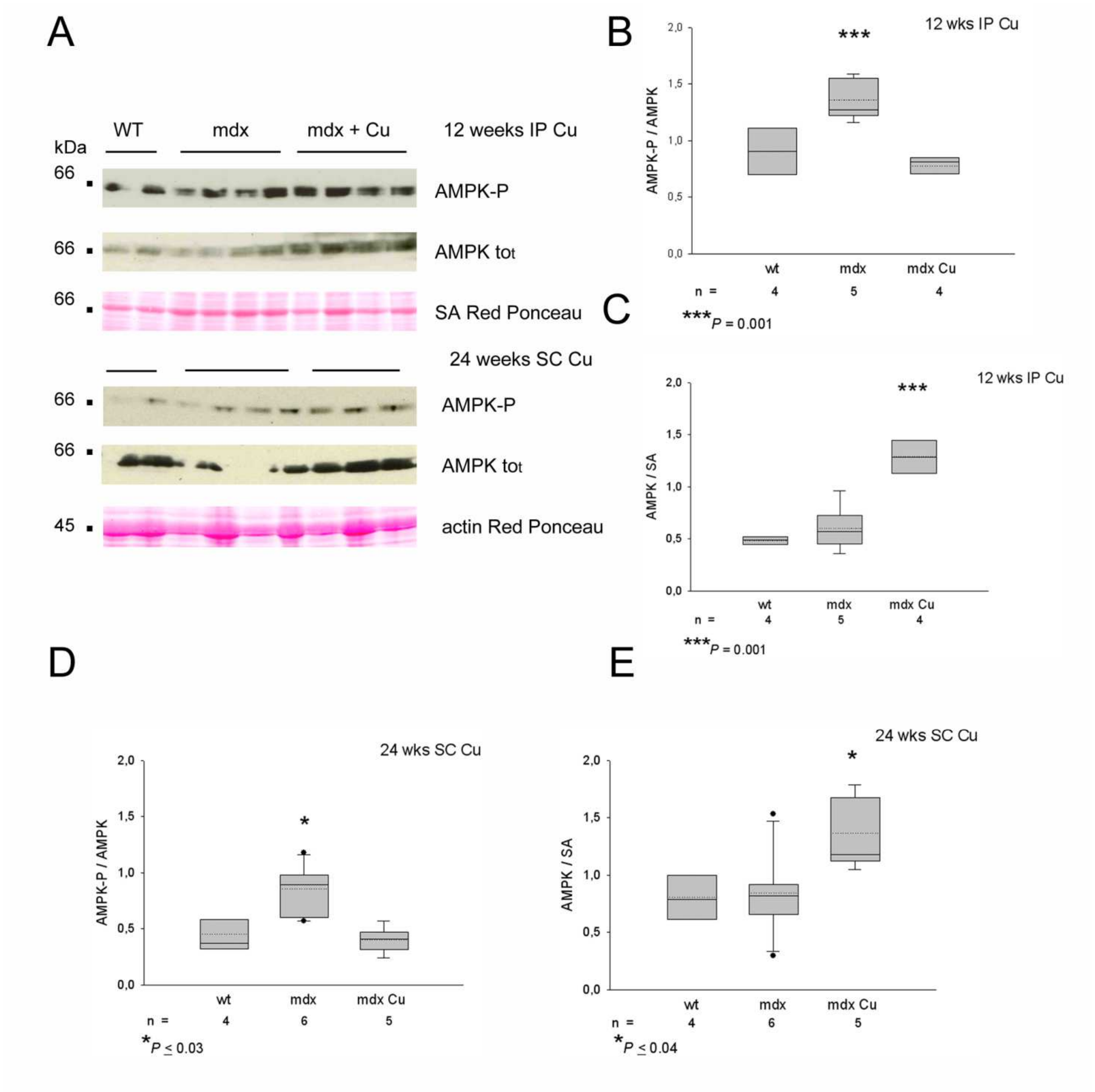

Figure S10: Curcumin effects on diaphragm AMPK-P and AMPK total protein levels.

A) Representative Western blots of diaphragm homogenates from wild type (wt) and mdx mice treated for 12-24 weeks with vehicle or curcumin (+Cu) by intraperitoneal (IP, upper panels) or subcutaneous (SC, lower panels) administration. Blots were stained with antibodies specific for phosphorylated and total AMPK. Loading is indicated by serum albumin (SA) or actin staining with Red Ponceau. Molecular weight standards are indicated on the left.

B– E) Box plots of densitometric values for phosphorylated and total AMPK ratio (B and D) and normalized total AMPK protein levels (C and E). Mean and median are indicated with a dotted and a solid line, respectively. n indicates the number of analyzed samples in each group. Asterisks indicate the presence of significant difference vs. all (ANOVA and post-hoc analyses).
